# Smoothing method for unit quaternion time series in a classification problem: an application to motion data

**DOI:** 10.1038/s41598-023-36480-y

**Published:** 2023-06-09

**Authors:** Elena Ballante, Lise Bellanger, Pierre Drouin, Silvia Figini, Aymeric Stamm

**Affiliations:** 1 Department of Political and Social Sciences, University of Pavia, Pavia, Italy; 2 BioData Science Unit, IRCCS Mondino Foundation, Pavia, Italy; 3 Department of Mathematics Jean Leray, UMR CNRS 6629, Nantes University, 44322 Nantes, France; 4 Department of Research and Development, UmanIT, Nantes, France

**Keywords:** Biomedical engineering, Mathematics and computing, Applied mathematics, Computational science, Scientific data

## Abstract

Smoothing orientation data is a fundamental task in different fields of research. Different methods of smoothing time series in quaternion algebras have been described in the literature, but their application is still an open point. This paper develops a smoothing approach for smoothing quaternion time series to obtain good performance in classification problems. Starting from an existing method which involves an angular velocity transformation of unit quaternion time series, a new method which employ the logarithm function to transform the quaternion time series to a real three-dimensional time series is proposed. Empirical evidences achieved on real data set and artificially noisy data sets confirm the effectiveness of the proposed method compared with the classical approach based on angular velocity transformation. The R functions developed for this paper will be provided in a Github repository.

## Introduction

The representation and analysis of the motion of human body is a research subject which has been constantly expanding with the increasing use of sensors. In time series analysis, smoothing is a fundamental step in real world applications, especially when sensors are involved, because a certain amount of noise is captured. The presence of noise can lead to unstable results or even wrong conclusions when data are analyzed and classification or clustering algorithms are applied.

In this paper, data registered by a motion sensor called MetaMotionR (MMR), from Mbientlab, are analysed. The sensor measures the spatial orientation of the hips and stores it as a quaternion time series. The motion of the hip joint is registered under two different conditions: natural walking and a walking made difficult by a knee immobilizer orthosis to simulate a walking impairment due to Amyotrophic Lateral Sclerosis (ALS), Multiple Sclerosis (MS) and other neurodegenerative diseases.

In this context, different smoothing techniques for quaternion time series are reviewed. The smoothing technique proposed in^1^ has been selected for its simplicity of implementation and its power in making available all the techniques developed in Euclidean spaces. On the basis of this method, a new technique is proposed and compared with the previous one on real and artificially noisy data to understand how the influence of the level of the noise affects the performance of a smoothing methods.

The rest of this paper is organized as follows: “Quaternion time series smoothing methods” section reviews the literature related to smoothing 1D quaternion time series and introduces our proposal. In “Description of the differences between approaches” section a theoretical comparison of some of the methods is presented. “Experimental results” section shows the experimental settings and results of the quaternion wavelet smoothing of real and noisy data in terms of classification performance. In “A real application: data on a human behaviour study” section the application of the proposed method to a real dataset related to a human behaviour study is described. Conclusions and further ideas for research are summarized in “Conclusions” section.

## Quaternion time series smoothing methods

We are interested in smoothing methods suitable for one-dimensional quaternion-valued signals. Let $f\in L^2({\mathbb {R}},{\mathbb {H}})$ be a signal, where ${\mathbb {H}}$ is the quaternion space (an introduction to quaternions and quaternion time series is described in Section 2 of Supplementary Material).

Most of the existing smoothing methods for this type of signal are generalizations of classical smoothing techniques originally introduced for Euclidean spaces: the Fourier transform, spline functions, and wavelets. These methods have been adapted to quaternion time series in different ways: spline functions are often used in quaternion algebras to interpolate signals^2,3^, while there are no examples of applications where splines are directly applied to smoothing signals.

Regarding the Quaternion Wavelet Transform (QWT), extensive reviews can be found in^4^ and^5^.

In^6^, naïve approach to smooth each component of the quaternions independently is described. It is well known in the literature that different isomorphisms between ${\mathbb {H}}$ and other spaces characterized by specific properties can be deployed to smoothing. More precisely the application of wavelet methods to quaternion time series is described in^7^, where Clifford wavelets and a Clifford multiresolution analysis are introduced. The application to quaternion time series is possible because ${\mathbb {H}}$ is isomorphic to the Clifford Algebra *Cl(0,2)*. The author limited his considerations to the theoretical statement of the method and the definition of the Haar wavelet.

In^8^, the idea of Mitrea was explored in the context of image analysis, and the Haar wavelet was applied to biomedical data (tomography images) written in terms of quaternions and compressed via wavelets.

The idea of matrix-valued wavelets (MVWs) was explored by Ginzberg and Walden in^9,10^, demonstrating that the wavelets defined in the previous literature^11,12^ are trivial: new matrix-valued wavelets, using the isomorphism between ${\mathbb {H}}$ and the space of matrices ${\mathbb {R}}^{4x4}$ with quaternion-structure conditions on the coefficients, are proposed. The isomorphism is defined as in Eq. (1).

1 $$\begin{aligned} q=w+x{\textbf{i}}+y{\textbf{j}}+z{\textbf{k}} \mapsto \begin{bmatrix} w &{}\quad -x &{}\quad -y &{}\quad -z\\ x &{}\quad w &{}\quad -z &{}\quad y \\ y &{}\quad z &{}\quad w &{}\quad -x\\ z &{}\quad -y &{}\quad x &{}\quad w \end{bmatrix} \end{aligned}$$

Adopting Eq. (1), quaternion-structured MVWs and hence quaternion wavelets are designed. The MATLAB code to compute the wavelet filter coefficients was presented, but the code to perform a wavelet analysis on quaternion signals is not provided.

In^10^ an application of MVWs to a simulated quaternion time series is described. Fletcher extended this work^13^ adding new wavelet filters of different lengths and explained how to arrange the filters in a matrix for analysing images, applying it to the analysis of a colour vector image.

In^14^ a different approach to analysing a quaternion signal is presented, with multi-resolution techniques, based on second generation wavelet transform. The quaternion lifting scheme is defined as follows: the input data set is split into two disjoint sets of even and odd indexed samples; samples with odd indices are predicted based on the sample with even indices, using the SLERP or SQUAD methods for quaternion time series (more details are in Section 2 of Supplementary materials). Next, the input value with the odd index is replaced by the offset (difference) between its value and its prediction. The outputs are updated, so that coarse-scale coefficients have the same average value as the input samples. This step is necessary for the stability of the wavelet transform.

In this procedure, the wavelet function used can be reconstructed, but it is not necessary for the computation.

Another approach to quaternion signal smoothing through wavelets is described in^1^, resorting the methods explored in^15^ and^16^ for the application of the Fourier transform to quaternion signals. The analysis is now focused on unit quaternion time series, where each element of the time series is an object in the space of unit quaternions ${\mathbb {H}}_1 \subset {\mathbb {H}}$, i.e. the space of unit norm quaternions.

The underlying idea is that if a unit quaternion time series is smooth, the changes of the angular velocities should be small^1^. With this rationale, the smoothing process can be applied in the angular velocity space. As the angular velocities are in three-dimensional Euclidean space, all the real wavelet techniques, and even more, in general, all smoothing techniques for Euclidean spaces can be deployed. After the smoothing process, the unit quaternion time series are reconstructed.

In order to obtain angular velocities without employing derivatives, the following approximation formulas are used. Given a unit quaternion time series $q_1,\ldots ,q_N$ and the time step *h* at which they were measured, the angular velocity is approximated as follows (see Eq. (4) for the definition of quaternion logarithm):

2 $$\begin{aligned} {\textbf{v}}_i=\frac{log(q_i^{-1}q_{i+1})}{h}, \, i=1,\ldots ,N-1. \end{aligned}$$

With these approximate angular velocities $\tilde{{\textbf{v}}}_1, ..., \tilde{{\textbf{v}}}_N$, the quaternion time series is reconstructed as (see (5) for the definition of quaternion exponential):

3 $$\begin{aligned} \tilde{q_i}=q_1 \prod _{j=2}^{i} \exp (\tilde{{\textbf{v}}_j}h), \, i=1,\ldots ,N-1. \end{aligned}$$

Following this idea, our proposed approach considers the logarithm transformation to go from ${\mathbb {H}}_1$ to its tangent space ${\mathbb {R}}^3$. The logarithm of a unit quaternion time series is a time series in 3-dimensional Euclidean space defined as follows:

4 $$\begin{aligned} \log (q)=\left( \frac{x}{|{\textbf{v}}|}\arccos \left( \frac{w}{|q|}\right) ,\frac{y}{|{\textbf{v}}|}\arccos \left( \frac{w}{|q|}\right) , \frac{z}{|{\textbf{v}}|}\arccos \left( \frac{w}{|q|}\right) \right) \in {\mathbb {R}}^3 \end{aligned}$$

The idea is to employ suitable smoothing process to each component of the logarithm of the quaternions and then compute the unit quaternions by taking the quaternionic exponential, as follows:

5 $$\begin{aligned} \exp (q)=\exp (w)(\cos (|{\textbf{v}}|),\frac{x}{|{\textbf{v}}|}\sin (|{\textbf{v}}|),\frac{y}{|{\textbf{v}}|}\sin (|{\textbf{v}}|),\frac{z}{|{\textbf{v}}|}\sin (|{\textbf{v}}|)) \end{aligned}$$

where the *w* component of the logarithm is always 0.

Note that the logarithm is defined on a sufficiently small neighbourhood of zero, the exponential is indeed globally defined, but is only bijective on a sufficiently small neighbourhood of zero. Hip motions are small in amplitude, so this issue should not affect our application.

The logarithm transformation is smoother with respect to the angular velocity and it has some intrinsic differences that will be explored in “Description of the differences between approaches” section. These differences in the definition of the transformed space, where the smoothing is performed, affect the performance in classification tasks in ways that will be described in “Experimental results” section.

## Description of the differences between approaches

Firstly, we are interested in the comparison of the images of the two transformations involved.

The quaternionic logarithm function, for a unit quaternion, is $f: {\mathbb {H}}_1 \mapsto {\mathbb {R}}^3$ such that $q=(w,x,y,z) \mapsto \log (q)=\frac{(x,y,z)}{|(x,y,z)|} \arccos (\frac{w}{|q|}) \in {\mathbb {R}}^3$, where, for ${\textbf{v}}=(x,y,z)$, write $\frac{{\textbf{v}}}{|{\textbf{v}}|}$ for the vector of unit norm in ${\mathbb {R}}^3$ in the same direction as ${\textbf{v}}$ and $\arccos (w/|q|)\in [0, \pi ]$.

As a consequence, $Image(f)=\{{\textbf{v}}\in {\mathbb {R}}^3 : |{\textbf{v}}|\le \pi \}$ is the ball of radius $\pi$ in ${\mathbb {R}}^3$.

Given a unit quaternion time series $q_1,\ldots ,q_N$, the angular velocities are approximated as in Equation (2): ${\textbf{v}}_i=\frac{log(q_i^{-1}q_{i+1})}{h}$, where $q_i^{-1}q_{i+1}\in {\mathbb {H}}_1$. Therefore, the same considerations can be applied to the numerator and $Image(f)=\{{\textbf{v}}\in {\mathbb {R}}^3: |{\textbf{v}}|\le \frac{\pi }{h}\}$ is the ball of radius $\frac{\pi }{h}$ in ${\mathbb {R}}^3$. In the theoretical framework, the angular velocity is calculated as a derivative and, in the limit for *h* that goes to 0, the image of the transformation is all of ${\mathbb {R}}^3$. Since in our application the angular velocity is approximated, *h* is a small positive constant and the image of the transformation is a ball in ${\mathbb {R}}^3$ with a radius larger than that with the logarithm transformation.

To further develop this comparison, we consider the geometric interpretation of the transformations involved: angular velocity and logarithm.

The logarithm function applied to a unit quaternion *q*, gives the point corresponding to *q* in the tangent space at the identity quaternion. So when we take the logarithm of a quaternion time series, we obtain a series lying entirely in that one specific tangent space.

The angular velocity transformation $log(q_i^{-1}q_{i+1})$ gives the point in the tangent space at $q_i$ corresponding to $q_{i+1}$. As a consequence, the corresponding time series in ${\mathbb {R}}^3$ is a collection of points lying in tangent spaces at different points.

Another critical issue that must be taken into account is that in the space ${\mathbb {H}}_1$ of unit quaternions, the product is not commutative.

As is well known, the formula $\exp (p)\exp (q)=\exp (p+q)$ does not hold in general when *p* and *q* do not commute. In this case, the Cambell-Baker-Hausdorff formula^17^ for the product of two non-commuting exponentials is applied and in the general case it provides an infinite correction term within the right-hand side of the exponential.

The problem was exactly solved for rotational data in *SO*(3)^18^ and in *SU*(*N*)^19^ (note that *SU*(2) is isomorph to ${\mathbb {H}}_1$).

An exact formula to determine the value of the quaternion $\alpha$ such that $\exp (p)\exp (q)=\exp (\alpha )$ is stated in^20^.

## Experimental results

This section will describe how the different smoothing methods and transformations affect the classification. All the Tables reporting the detailed results commented in “Classification results” section can be found in Section 1.2 of Supplementary materials.

### Data description

The original data set consists of 54 unit quaternion time series of 101 observations each. The time goes from 0 to 100 (%) in steps of 1%. The data were recorded by a wearable motion sensor called MetaMotionR (MMR) an Inertial Measurement Unit (IMU) from Mbientlab. It is a device that combines a three-axis accelerometer, a gyroscope, and magnetometer, to determine its orientation in the form of a unit quaternion. It is worn at the level of the hip to measure the angle of rotation of the hip during walking movements, at a frequency of 100 Hz. The signal captured by a motion sensor is periodic and composed of actual walking steps referred to as gait cycles. A gait cycle is defined as the sequence of movements performed by the body during the phase delimited by two successive contacts of a given foot with the ground. We therefore compute an average gait cycle, referred to as the individual gait pattern, by jointly aligning in time and pointwise averaging the segmented gait cycles.

Data related to 27 healthy subjects were collected under two different conditions. The first evaluation was made letting the subject perform a natural walking movement. Another record was made using a knee immobilizer orthosis to simulate a walking impairment.

To represent 3D rotations we choose a unit quaternion representation for convenience, as suggested in the literature on 3D rotation analysis^21^.

A unit quaternion represents a 3D rotation between a given object’s frame, or coordinate system (the IMU’s coordinate system), and a fixed coordinate system defined as the reference. We choose the first orientation observed of the Individual Gait Pattern (IGP) as the reference, and each unit quaternion of the IGP represents the rotation between this first orientation and the one observed at a given time. For this reason, in the original dataset, the first element of each time series is the quaternion (1 0 0 0), representing the identity rotation. We also processed the data in order to ’straighten’ the IGP, so that the first and the last element of the IGP are the identity rotation. In order to apply wavelet methods, the original time series is re-sampled to have 128 time points.

**Figure 1:**
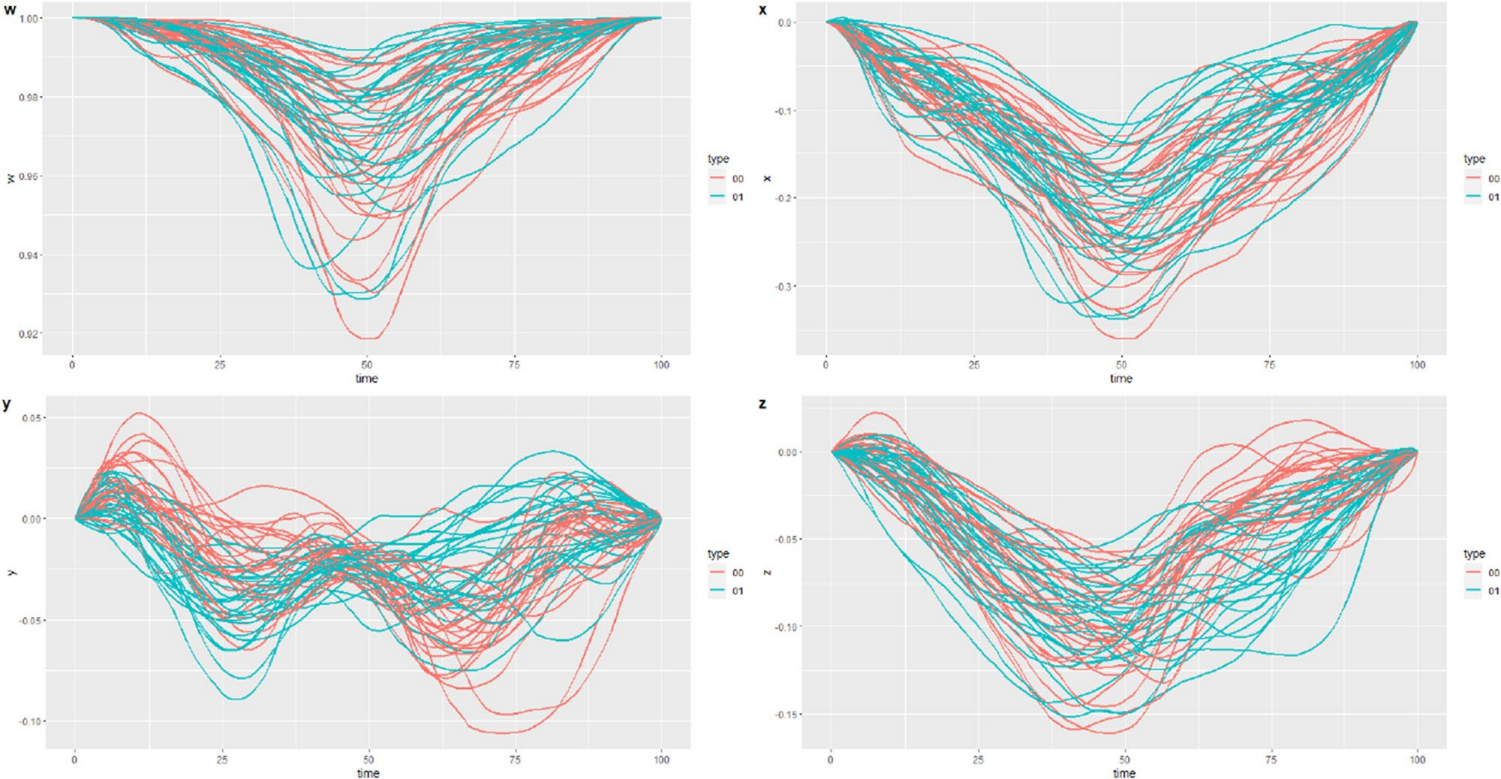
Component-wise representation of the individual gait pattern data.

Figure 1 depicts the component-wise representation of the individual gait pattern data; color represents the two conditions in which the data are collected and that will be used for the classification task: the natural walk and the hindered walk. Alternative representations are provided in Section 3 of Supplementary Materials. In order to understand the influence of noise on the performance of the different smoothing methods, we applied the methods described in “Methods and experimental settings” section to data to which different levels of noise had been added. We generated these noisy data sets by adding Gaussian noise to the logarithm of the curves in the original data set^22^. The quaternion time series were transformed to ${\mathbb {R}}^3$ through the logarithm transformation and then Gaussian noise was added independently to each component. Consider the observation $X_{i,k}$, where *i* corresponds to the *i*-th subject and *k* correspond to the *k*-th component of the multidimensional time series, *m*(*t*) identifies the median line, and $\epsilon$ is a Gaussian error term:

$$\begin{aligned} X_{i,k}=m_k(t)+\epsilon _k(t), \quad Cov(\epsilon _k(s),\epsilon _k(t))=C(s,t), \quad \forall i=1,\ldots ,N, \, \forall i=k,\ldots ,L \end{aligned}$$

where $Cov(\epsilon _k(s),\epsilon _k(t))=C(s,t)$ is generated as an exponential-like covariance function with two parameters:

6 $$\begin{aligned} C(s,t)=\alpha e^{-\beta |s-t| }. \end{aligned}$$

Different degrees and types of noise were simulated by varying the parameters $\alpha$ and $\beta$:

- $\alpha =0.001$, $\beta =0.01$. Low noise, moderately correlated.
- $\alpha =0.01$, $\beta =0.001$. Moderate noise, highly correlated.
- $\alpha =0.01$, $\beta =0.01$. Moderate noise, moderately correlated.
- $\alpha =0.01$, $\beta =0.1$. Moderate noise, weakly correlated.
- $\alpha =0.1$, $\beta =0.01$. High noise, moderately correlated.

The datasets obtained are visually represented in Section 1.1 of Supplementary Materials.

### Methods and experimental settings

Wavelet smoothing method with Fourier and spline smoothing, each one embedded in one of the two transformations from ${\mathbb {H}}_1$ to ${\mathbb {R}}^3$, are compared.

In order to smooth signals using wavelets, the discrete wavelet transform was applied, with soft thresholding in its generalized sense for multidimensional signals^23^:

$$\begin{aligned} {\bar{\textbf{w}}}={\left\{ \begin{array}{ll} {\textbf{0}}, &{} \text {if } ||{\textbf{w}}||\le t_p;\\ \left( 1-\frac{t_p}{||{\textbf{w}}||}\right) {\textbf{w}}, &{} \text {if } ||{\textbf{w}}||> t_p; \end{array}\right. } \end{aligned}$$

where ${\textbf{w}}$ are the *p*-dimensional vectors of the detail coefficients of the DWT. The chosen threshold was the universal threshold as generalized in^23^: $t_p=\sigma \sqrt{3\log (N)}$ where $\sigma$ is the standard deviation of the noise. Since $\sigma$ is generally unknown in practical situations, it must be estimated following the idea described in^24^, where the Median Absolute Deviation (MAD) of the details coefficients was proposed ($MAD({\textbf{x}})=median(|x-median(x)|)$). The estimated standard deviation in the multidimensional case is:

$$\begin{aligned} {\hat{\sigma }}=\frac{MAD(\mathbf {{\textbf{d}}_1})}{0.6745} \end{aligned}$$

where $\mathbf {d_1}=\{d_{1,k}^i\}_{k,i}$ is the vector of detail coefficients obtained from the first level of decomposition of each component function (all pooled together).

The following mother wavelets and decomposition levels are considered:

- Mother wavelets: Haar, Daubechies 4 (d4), Daubechies 6 (d6), Daubechies 8 (d8), Daubechies 16 (d16), Least Asymmetric 8 (la8), Least Asymmetric 16 (la16), Least Asymmetric 20 (la20), Best Localized 14 (bl14), Best Localized 20 (bl20).
- Decomposition levels (DLs): from 1 to 6.

The Fourier smoothing was performed through a non-parametric regression smoothing using 20, 40 and 60 basis elements. No covariates and no roughness penalty was used.

Linear, cubic and quintic splines were employed with cross validated parameters for each curve. The number of knots considered is 71. The parameters selected are not optimal, because their optimization it is outside the scope of this paper.

For each combination of parameters, the smoothing process is evaluated in terms of classification performance. A *k*-Nearest Neighbours (*k*-NN) model is used to perform classification on the original and on the smoothed quaternion time series to select the smoothing method that removes noise while preserving the most important features that can distinguish between two groups.

The *k*-NN algorithm is a non-parametric classification method firstly developed in^25^. An observation is classified by a plurality vote of its neighbours, with the object being assigned to the class most common among its *k* nearest neighbours. Here, *k* is a positive integer, typically small, that we will set to the standard value of 5. The value of *k* is generally optimized based on data at hand, but that is outside the scope of this paper.

Being a distance-based algorithm, it is easily generalized to quaternion time series using the Dynamic Time Warping (DTW) distance^26^. For more details about DTW, see Section 2 of Supplementary Materials.

The results presented in the present paper are based on a cross validation exercise, where 5 folds are defined to obtain stable results working with a small sample size.

For each series in the test fold, the distances from the series in the training fold are computed. The 5 nearest time series in the training set are considered and the majority label is assigned to the tested series.

The results are evaluated in terms of accuracy, expressed in terms of percentage of correct classified observations, coupled with AUC (area under the ROC curve). The accuracy measures are computed on the 5 folds and summarized in terms of averaged values.

To increase the robustness of the conclusions, linear regression models were studied to model the influence of the transformations and of the choice of smoothing methods on the performance indices. Each level of noise described in “Data description” section was simulated ten times and the original data were simulated adding a minimal noise setting $\alpha =0.0001$ and $\beta =0.0001$. The accuracy and the AUC were evaluated and considered as target variables, and smoothing method and type of transformation were considered as covariates.

All the computations were performed using the R software (R Core Team (2017)), the figures are generated with the ggplot2 package (v3.3.3; Wickham, 2016) and the plotly package (Plotly Technologies Inc. Collaborative data science. Montréal, QC, 2015. https://plot.ly.). The R functions developed for this work will be provided in a Github repository.

### Classification results

The performances reached by *k*-NN on the original individual gait pattern data set have an accuracy of 0.8200 and an AUC of 0.9149.

Applying a smoothing process to the data after the logarithm transformation, for all the methods and all the choices of parameters, the accuracy is 0.8100 and almost all the AUCs are 0.8531, with small differences for some combinations of the parameters. Considering the angular velocity transformation, the performances are lower than both the original data and the logarithm smoothing process.

This shows that the smoothing process does not improve the classification performance when the curves considered are already nearly smooth. Instead, in some cases, the performances are lower, which seems to suggest that the smoothing process removes some important features in the data that are already exploitable.

**Figure 2:**
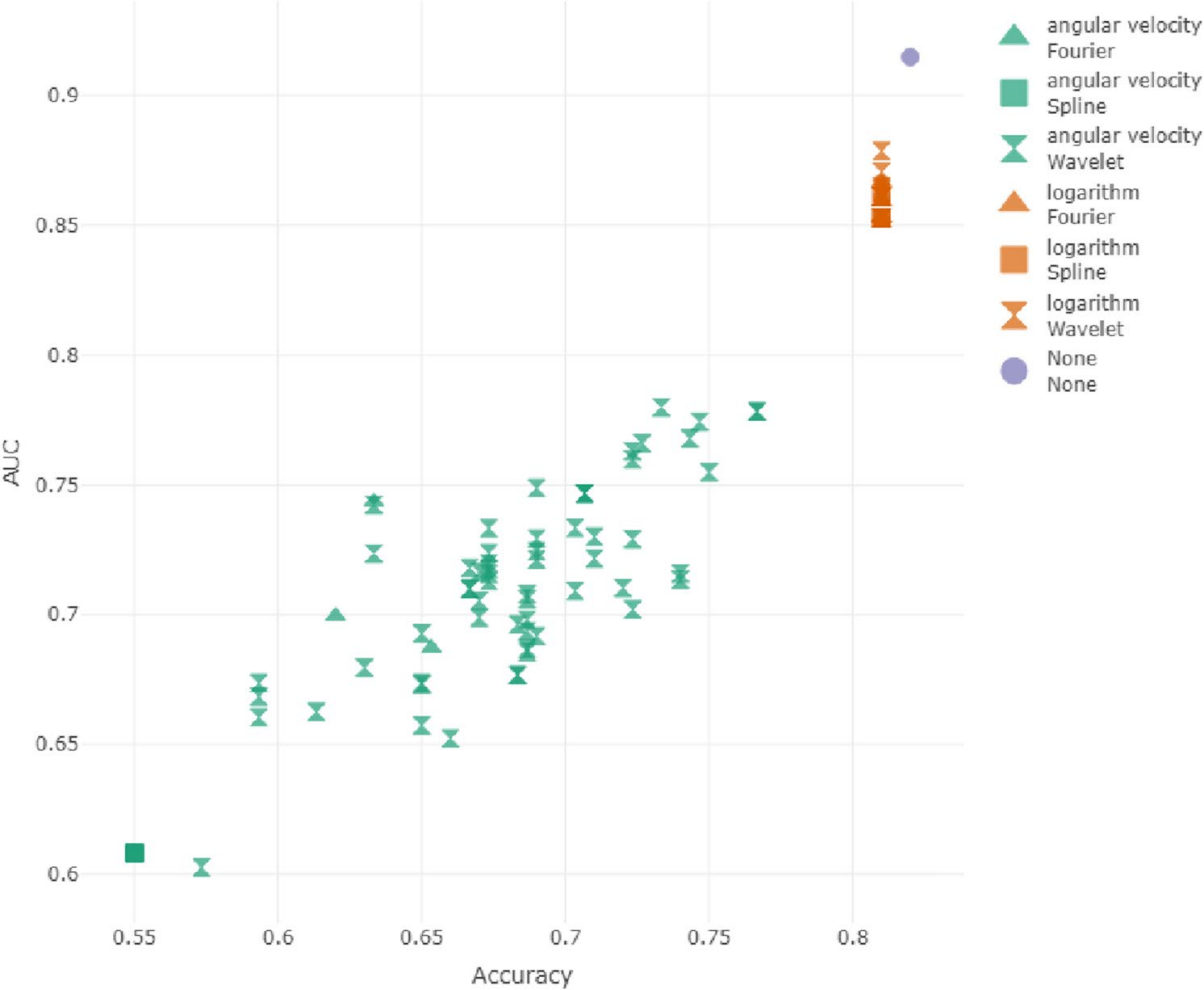
Results on original data. Performances of the different methods are evaluated in terms of accuracy and AUC. The shape distinguishes between Fourier, spline or wavelet smoothing methods and colours distinguish between logarithm and angular velocity transformations.

The comparison between angular velocity methods and the logarithm shows that when smooth functions are involved, the logarithm better preserves the characteristics of the curves, as in Fig. 2. An explanation for this result could be that the logarithm transformation is smoother than the angular velocity, which presents a higher variability also for regular curves.

**Figure 3:**
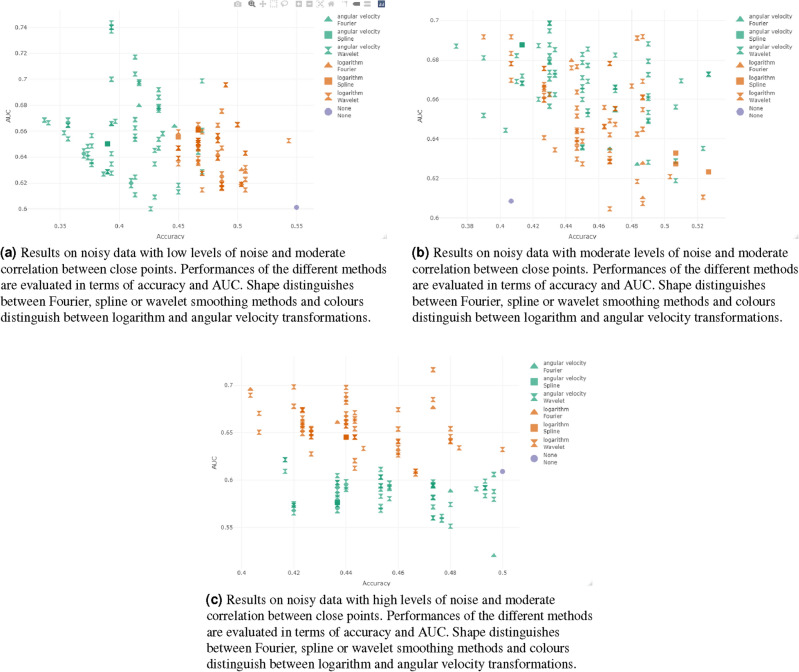
Results obtained in the classification using noisy datasets with a fixed value of the autocorrelation ($\beta =0.01$), increasing the value of the variance parameter ($\alpha =0.001$, $\alpha =0.01$, $\alpha =0.1$).

#### Classification results on noisy data: analysis of variance at fixed autocorrelation

Now consider the performances reached with noisy data sets as defined in “Data description”. We start with a fixed value of the autocorrelation ($\beta =0.01$), increasing the value of the variance parameter ($\alpha =0.001$, $\alpha =0.01$, $\alpha =0.1$).

Consider at first the data set generated with $\alpha =0.001$ and $\beta =0.01$. We are introducing low levels of noise (the noise has low variance) and a moderate correlation between the nearest points.

The performances reached without any smoothing have an accuracy of 0.550 and an AUC of 0.601. The best method can be identified as the wavelet smoothing method with different combinations of parameters: wavelet d6 with 4 in terms of accuracy (accuracy = 0.5433 and AUC = 0.6527) and wavelet d4 with 5 and 6 decomposition levels in terms of AUC (accuracy = 0.49 and AUC = 0.6958).

The angular velocity method achieves poorer results in terms of classification accuracy for all the smoothing functions and choices of parameters (accuracy$\le$0.47) as in Fig. 3a. The highest values of AUC are reached with wavelet d16 with 1 decomposition level (accuracy = 0.3933 and AUC = 0.7434).

Note that almost all the smoothing methods and transformation yield higher AUC but lower accuracy. The only method competitive with the non-smoothed data set is the best of the logarithm transformation.

Now consider the data set with moderate noise variance and moderate correlation ($\alpha =0.01$ and $\beta =0.01$). Data classification without any smoothing obtains an accuracy of 0.407 and an AUC of 0.608.

In this case the logarithm transformation performs similarly to the angular velocity and it is difficult to identify the best method as in Fig. 3b. Almost all the smoothing methods obtain better results than the non-smoothed data set.

Now consider the noisy data set generated with high noise variance and moderate correlation between close points ($\alpha$=0.1 and $\beta$=0.01). Data classification without any smoothing reaches an accuracy of 0.5 and an AUC of 0.609.

One method reaches better results than the original data classification in terms of the AUC, but with the same accuracy (wavelet la20 with 3 decomposition levels, accuracy=0.5 and AUC=0.6322). Higher values of AUC are reached with lower levels of accuracy: for this reason it is difficult to identify the best method. The logarithm transformation seems to obtain better results in terms of AUC than does the use of the angular velocity, with similar values of accuracy. See Fig. 3c.

**Figure 4:**
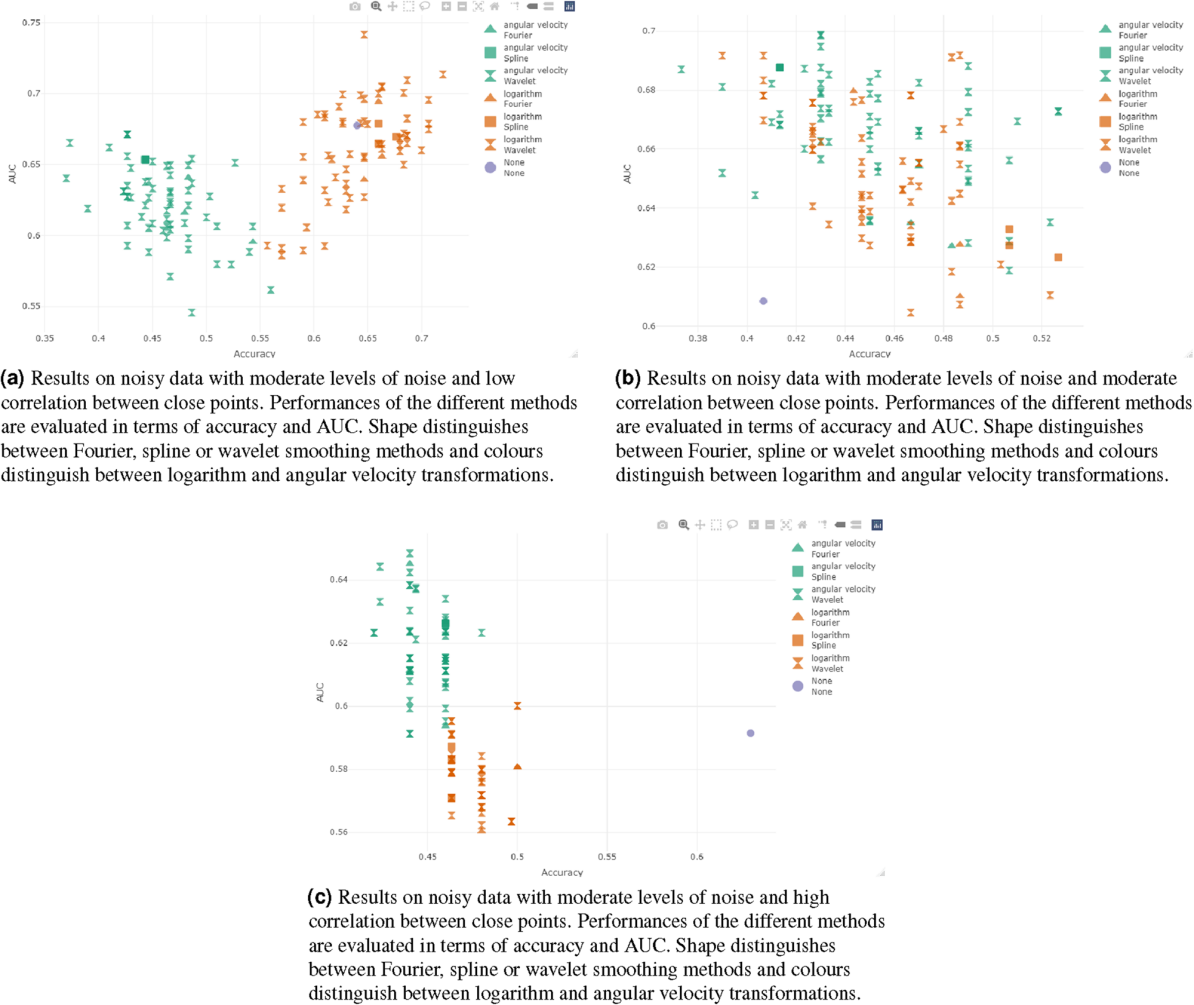
Results obtained in the classification using noisy datasets generated with a fixed value of variance ($\alpha =0.01$), varying the value of the autocorrelation parameter ($\beta =0.001$, $\beta =0.01$, $\beta =0.1$).

#### Classification results on noisy data: analysis of autocorrelation at fixed variance

Consider the performances reached with noisy data sets generated with a fixed value of variance ($\alpha =0.01$), varying the value of the autocorrelation parameter ($\beta =0.001$, $\beta =0.01$, $\beta =0.1$).

Considering the data set with moderate noise variance ($\alpha =0.01$) and an high correlation between close points ($\beta =0.001$), we obtain that data classification without any smoothing reaches an accuracy of 0.630 and an AUC of 0.591.

In this data set the angular velocity performs better than logarithm in terms of AUC, but worse in terms of accuracy. Both the transformations with all the methods obtain worse performances than the raw data classification and no smoothing is suggested, as in Fig. 4a.

In general we confirm that when noise levels are low, the smoothing process is not necessary and it increases the risk of removing important features of the data set.

Now consider the data set with moderate noise variance and moderate correlation ($\alpha =0.01$ and $\beta =0.01$). As seen before, data classification without any smoothing obtains an accuracy of 0.407 and an AUC of 0.608.

Almost all the smoothing methods obtain better results than the non-smoothed data set, as in Fig. 4b.

Now consider the noisy data generated with a moderate noise variance and low correlation between close points ($\alpha =0.01$ and $\beta =0.1$). Data classification without any smoothing reaches an accuracy of 0.640 and an AUC of 0.678.

A lot of smoothing methods reach better results than the original data classification, but only if we consider a smoothing transformation. The angular velocity transformation seems to have lower results. The best result in terms of accuracy is reached by wavelet d4 with 1 decomposition level (accuracy=0.7200, AUC=0.7136). In terms of AUC the best method is wavelet la8 with 4 decomposition levels, accuracy=0.6467 and AUC=0.7416 (see Fig. 4c).

### Final results

**Figure 5:**
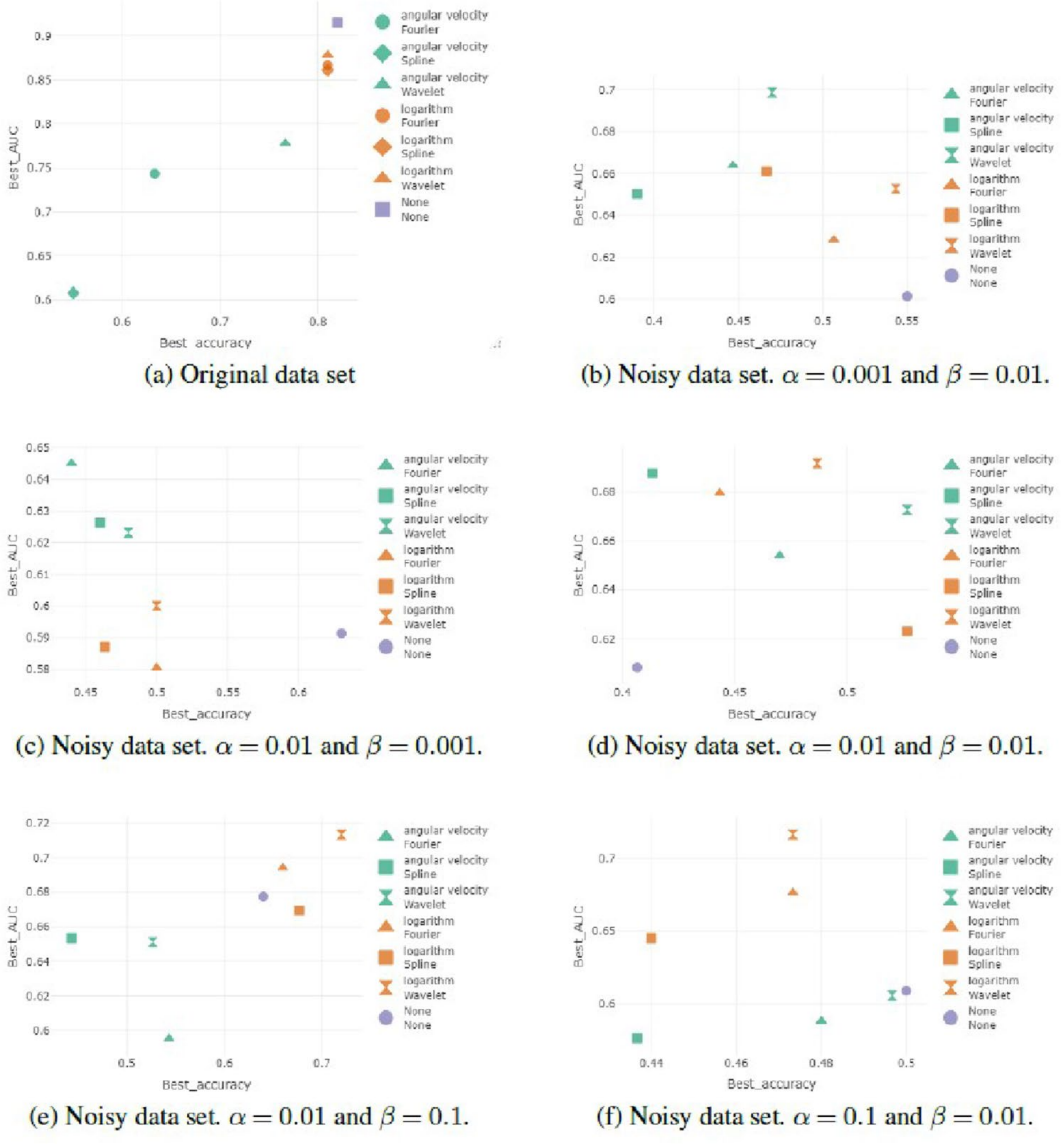
For each method (Fourier, spline and wavelet) and transformation (logarithm and angular velocity) the best result is presented, where the best result is identified by using the sum of the accuracy and the AUC. Shape distinguishes between Fourier, spline or wavelet methods and colours distinguish between logarithm and angular velocity transformations.

The influence of noise is clear: when the curves are nearly smooth, the smoothing methods can not improve in the classification, whereas when we introduce noise (both in terms of high variance and low autocorrelation), the need for applying smoothing methods becomes clear and the performance can be improved by the process, as we can see in Fig. 5.

In order to confirm the validity of the proposed method, a linear regression analysis of the accuracy and the AUC has been performed, where the influence of the transformation function and the smoothing method is evaluated. Ten data sets have been generated for each combination of parameters $\alpha$ and $\beta$, as defined in Eq. (6), and the original data set is simulated with the parameters $\alpha =\beta =0.0001$. The covariates of the three models are:

- The variable ’transformation’ indicates if the transformation function is angular velocity or logarithm. It is a factor variable with reference value ’angular velocity’ (two levels).
- The variable ’smoothing_method’ indicates if the smoothing method is Fourier, spline or wavelet. It is a factor variable with reference value ’Fourier’ (three levels).
- Par alpha and par beta correspond to the noise parameters as defined in Eq. (6) and are numerical variables.

The target variables of the three models are the accuracy and the AUC. The logit transformation is applied to each of the target variables to transform the range from [0,1] to $(-\infty ,+\infty )$. This produces a larger range of values than the other common transformations. Because the target variables still do not satisfy the normality assumption, and common transformations do not solve the problem, a bootstrap procedure is applied to obtain the coefficients and confidence intervals.

The results regarding the model for accuracy outcome are summarized in Table 1. As the results for AUC are similar to the accuracy ones, they are shown only in Section 1.3 of Supplementary Materials. The ANOVA tables for the linear models are also presented in Section 1.3 of Supplementary Materials.

**Table 1:** Linear regression model for accuracy target variable with a bootstrap procedure.

Variables	Coefficients	stdev	CI	
Intercept	0.088	0.020	(0.051, 0.125)	*
Transformation logarithm	0.121	0.008	(0.106, 0.138)	*
Smoothing method spline	0.011	0.028	(−0.043, 0.068)	
Smoothing method wavelet	0.022	0.020	(−0.043, 0.068)	
Noise variance ($\alpha$)	$-$2.965	0.114	(−3.196, −2.756)	*
Noise autocorrelation ($\beta$)	$-$0.752	0.097	(−0.945, −0.562)	*

The smoothing methods (wavelet, spline and Fourier) do not seem to have a global impact on the quality of the smoothing process in terms of the classification performance: the coefficients of the wavelet and spline methods compared to the reference level (Fourier) are not significant. Instead, the coefficient related to the logarithm transformation with respect to the angular velocity transformation is significantly different from zero and positive. The results confirm the positive effects of the logarithm transformation on that target variable. We can also observe that the variance and autocorrelation parameters in the noise generation are significant, with negative coefficients. Higher levels of noise have a negative impact on the classification performances, as can be expected.

## A real application: data on a human behaviour study

In order to confirm the potential of our proposed method, this section reports the empirical evidence achieved on a highly noised dataset. The data considered are related to a behavioural study about small free-standing conversational groups. A sample of the dataset (called CongreG8) is available at the following link: https://zenodo.org/record/4537811 and it is described in^27^.

The dataset contains full-body motion data collected from free-standing conversational groups of three human with a newcomer that approaches the group with the intention of joining it.

**Figure 6:**
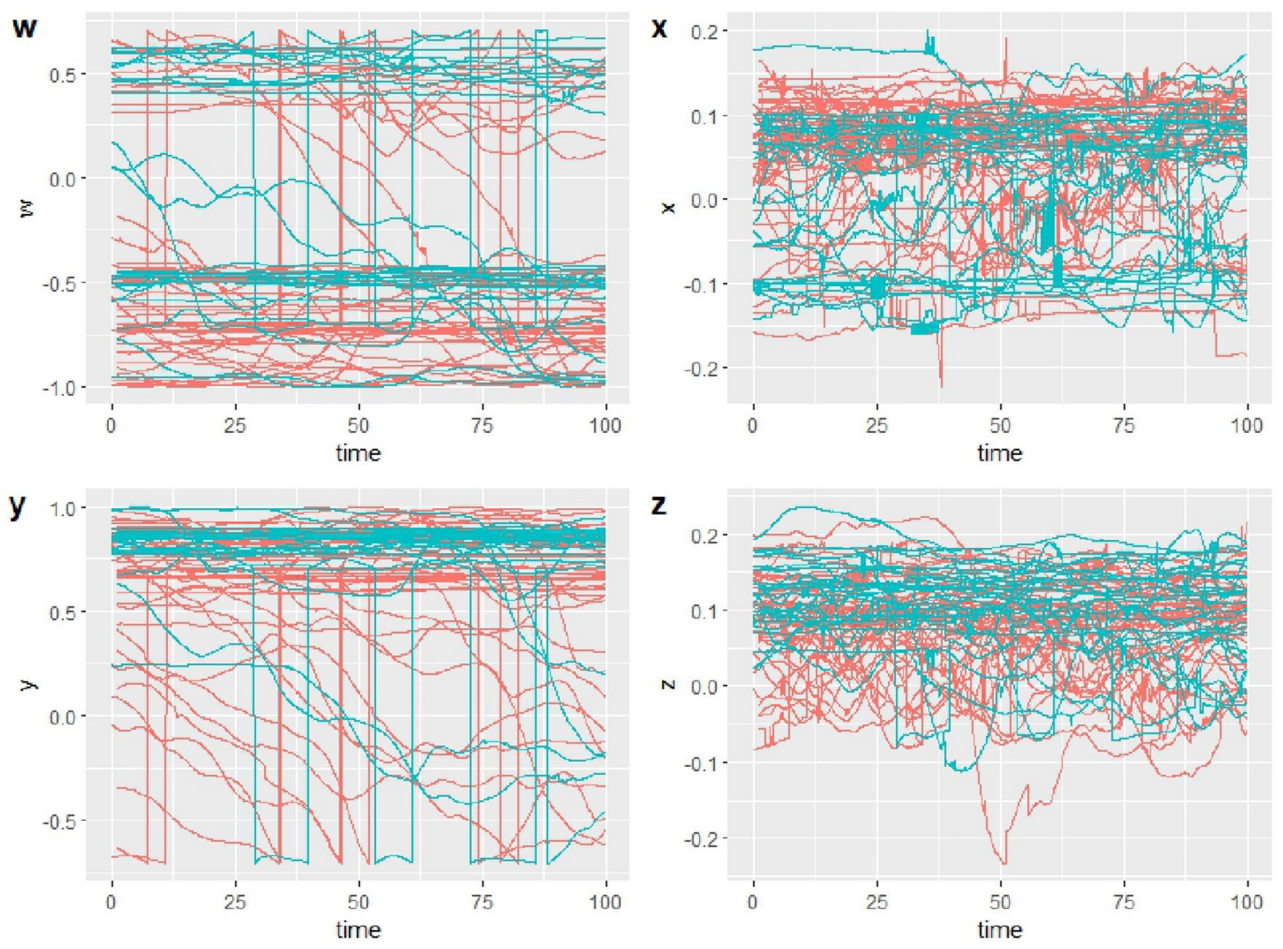
Component-wise representation of the quaternion time series of abdominal orientation. Blue lines represent subjects in groups with behaviour *Ignorance* and red lines represent subjects in groups with behaviour *Welcome*.

From the quaternion time series collected by 37 body markers, the skeleton was digitally reconstructed and different bones were tracked in terms of position and orientation. From these last set of time series we selected the abdominal orientation considering that it could be the one that better summarize and capture the personal attitude of the people in small groups with respect to the newcomer. We also sample one subject from each group to control correlation between subjects and computational time. Being of different lengths, each time series was cut to a standard length of 1024 that correspond to a temporal span of 8.5 seconds.

The target variable is related to the behaviour of the group, annotated by the authors as *Welcome* or *Ignore*.

After the preliminary selection, a dataset containing quaternion time series related to 80 subjects, 27 of which labeled as “Ignore” and 53 as “Welcome”, is obtained.

**Figure 7:**
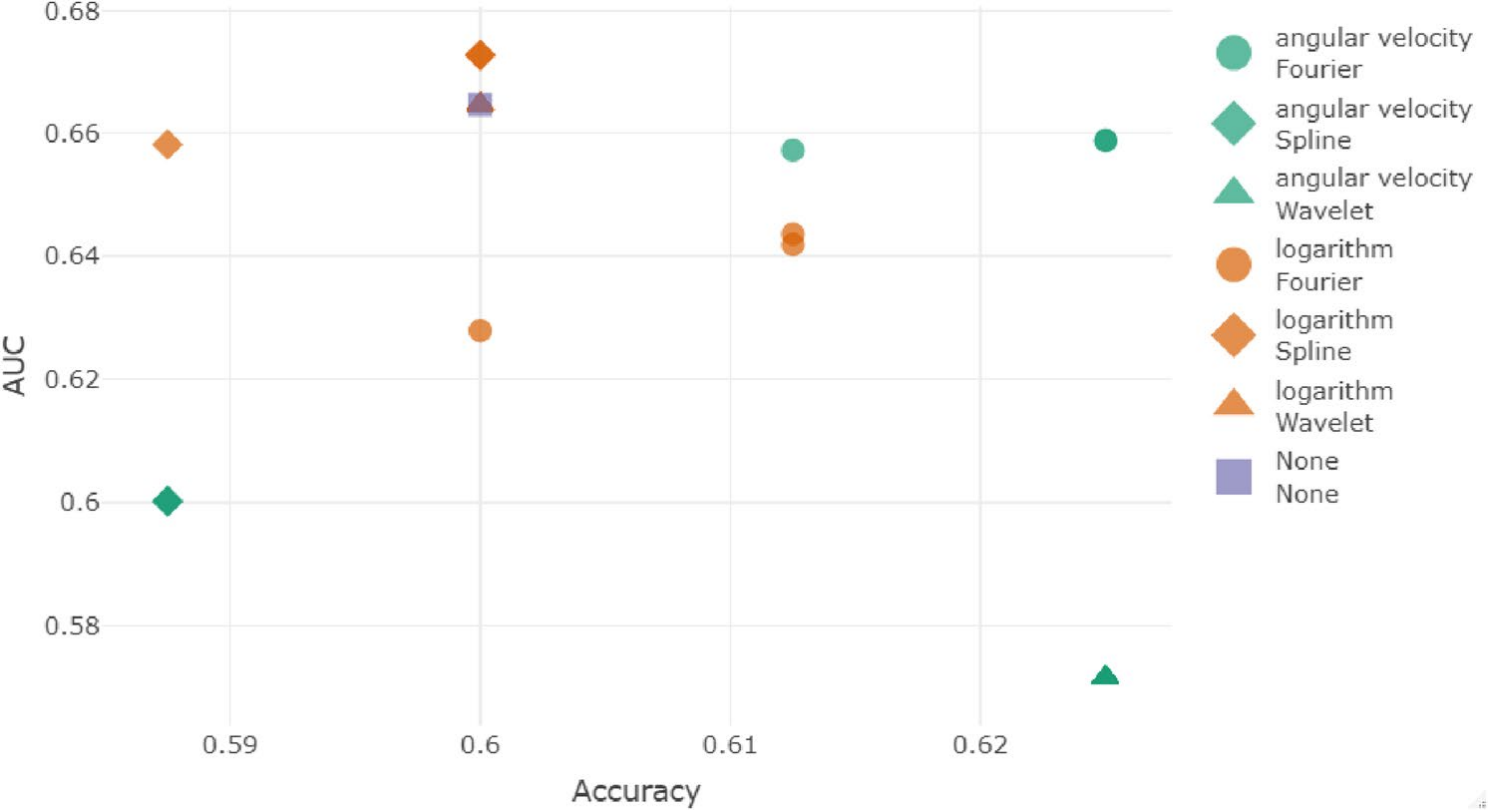
Results on real data evaluated in terms of accuracy and AUC. Shape distinguishes between Fourier, spline or wavelet smoothing methods and colours distinguish between logarithm and angular velocity transformations.

The final dataset is shown in Fig. 6. Alternative representations are provided in Section 3 of Supplementary Materials. The time series are extremely noised at a visual inspection and the discriminatory power is not clear. Figure 7 depicts the results obtained adapting the pipeline described in “Experimental results” section.

Performances of the different smoothing methods are evaluated in terms of classification performances measured as accuracy and AUC. In Fig. 7, shape distinguishes between Fourier, spline or wavelet smoothing methods and colours distinguish between logarithm and angular velocity transformations. The classification of data without the application of any smoothing method obtain and accuracy of 0.6 and an AUC of 0.665.

We observe that the accuracy is slightly improved by Fourier in logarithm method, Fourier in angular velocity and wavelets in angular velocity. This improvement does not correspond to an improvement of the AUC, that is lower than the original dataset classification one.

The best methods are cubic and quintic splines with logarithm transformation, that obtain the best results in terms of AUC. The accuracy is 0.6 as the original classification, but could be improved varying the threshold for classification (set as 0.5 by default).

We also observe that the application of different wavelets do not introduce any variability in the results. Wavelets applied with the logarithm method reach the same performances of the data without smoothing. Angular velocity methods show better results in terms of accuracy but much worse in terms of AUC.

We confirm the conclusion of the simulation study and of the regression methods: the best smoothing method seems to be data specific, and the logarithm transformation leads in general to better results than the angular velocity one. On the basis of the empirical evidence achieved on real data, better performances are reached in terms of AUC and further investigation about threshold selection is required to observe the improvement also in terms of accuracy. This leads us to improve in a further research study how threshold selection affects performances.

## Conclusions

This paper presents a new method to smooth unit quaternion time series.

This new method manages unit quaternions in a proper way, transforming the time series to an Euclidean space to take advantage of all the existing smoothing techniques. We compare wavelet methods with respect to Fourier and spline smoothing methods.

The results were evaluated in terms of their classification performance on a data set of unit quaternion time series describing walking cycles with a binary outcome variable. Five versions of this data set were created by adding noise to the original data to evaluate the influence of different degrees of noise on the smoothing process.

The results on the original data set and on the noisy ones confirm the need for applying smoothing techniques when the data are noisy and the opportuneness of deploying the proposed method (namely, using the logarithm transformation of unit quaternion time series) to obtain in general better results. Instead, we obtained no evidence about which one of the different smoothing techniques in ${\mathbb {R}}^3$ should be used, it seems to depend on the particular data set to be analyzed and should be evaluated on a case by case basis.

The application of the proposed method to a real noisy dataset confirms the conclusion of the simulated study. Further avenues of research include the application of different noise models to evaluate the influence of the particular nature of the data set, the application of other classification models, and a deeper analysis of the classical smoothing methods applied in this context. Furthermore, quaternion representation and visualization methods in R will be explored in depth. The approach described in this paper can be exploited in terms of the functional representation of quaternion time series, but this aspect needs further study.

The R functions developed for this work will be provided in a Github repository.

## Supplementary Information

Supplementary Information.

## Data Availability

The original datasets generated and analysed during the current study relateed to walking cycle application are available from the corresponding author on reasonable request. Instead, data analysed in “A real application: data on a human behaviour study” section are available in Zenodo repository at https://zenodo.org/record/4537811.
