# Supplementary material for: Smoothing method for unit quaternion time series in a classification problem: an application to motion data
Source: Sci Rep. 2023 Jun 9;13:9366. doi: 10.1038/s41598-023-36480-y (PMC10256761; doi:10.1038/s41598-023-36480-y)
Supplement: Supplementary file 1 — Supplementary Information. [file 41598_2023_36480_MOESM1_ESM.pdf]

Supplementary Information

1 Additional Figures and Tables

1.1 Simulated data description

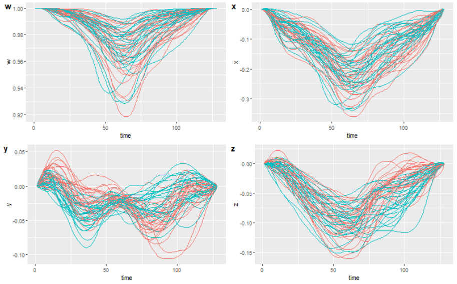

(a) Original data set

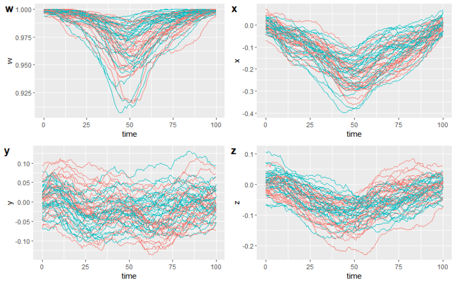

(b) Noisy data set. $\alpha = 0.001$ and $\beta = 0.01$.

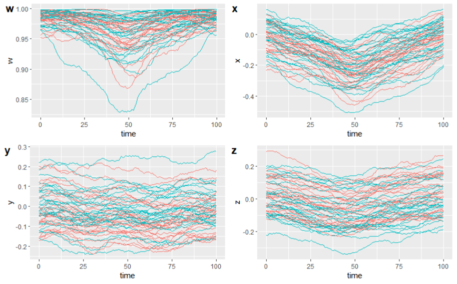

(c) Noisy data set. $\alpha = 0.01$ and $\beta = 0.001$.

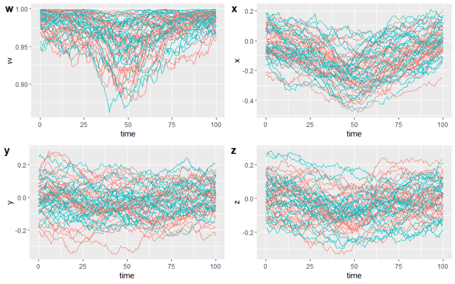

(d) Noisy data set. $\alpha = 0.01$ and $\beta = 0.01$.

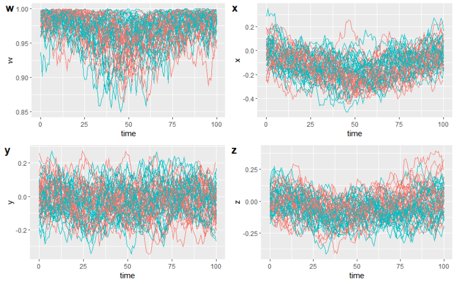

(e) Noisy data set. $\alpha = 0.01$ and $\beta = 0.1$.

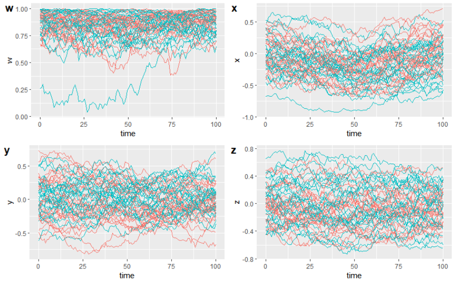

(f) Noisy data set. $\alpha = 0.1$ and $\beta = 0.01$.

Figure S1. Component-wise representation of the individual gait pattern data with the different levels of noise. The colour indicates which of the two conditions represents the curve.

	α	β	Noise
First noisy dataset	0.001	0.01	Low noise, moderately correlated
Second noisy dataset	0.01	0.001	Moderate noise, highly correlated
Third noisy dataset	0.01	0.01	Moderate noise, moderately correlated
Fourth noisy dataset	0.01	0.1	Moderate noise, weakly correlated
Fifth noisy dataset	0.1	0.01	High noise, moderately correlated

Table S1. Combination of parameters for the generation of the noisy data.

1.2 Detailed results of classification task on different simulated datasets

Method	Accuracy	AUC
Linear spline	0.8100	0.8611
Cubic spline	0.8100	0.8531
Quintic spline	0.8100	0.8531
Fourier 20 basis	0.8100	0.8667
Fourier 40 basis	0.8100	0.8531
Fourier 60 basis	0.8100	0.8531

Table S2. Original data, spline and Fourier smoothing methods, logarithm transformation

DLs	1	2	3	4	5	6
haar	0.8100	0.8100	0.8100	0.8100	0.8100	0.8100
d4	0.8100	0.8100	0.8100	0.8100	0.8100	0.8100
d6	0.8100	0.8100	0.8100	0.8100	0.8100	0.8100
d8	0.8100	0.8100	0.8100	0.8100	0.8100	0.8100
d16	0.8100	0.8100	0.8100	0.8100	0.8100	0.8100
la8	0.8100	0.8100	0.8100	0.8100	0.8100	0.8100
la16	0.8100	0.8100	0.8100	0.8100	0.8100	0.8100
la20	0.8100	0.8100	0.8100	0.8100	0.8100	0.8100
bl14	0.8100	0.8100	0.8100	0.8100	0.8100	0.8100
bl20	0.8100	0.8100	0.8100	0.8100	0.8100	0.8100

Table S3. Original data, accuracy, wavelet smoothing method, logarithm transformation

DLs	1	2	3	4	5	6
haar	0.8639	0.8651	0.8707	0.8787	0.8627	0.8627
d4	0.8531	0.8611	0.8611	0.8611	0.8611	0.8611
d6	0.8531	0.8531	0.8531	0.8611	0.8611	0.8611
d8	0.8531	0.8611	0.8611	0.8611	0.8531	0.8611
d16	0.8531	0.8531	0.8531	0.8531	0.8531	0.8531
la8	0.8531	0.8531	0.8611	0.8611	0.8611	0.8611
la16	0.8531	0.8531	0.8531	0.8531	0.8531	0.8531
la20	0.8531	0.8531	0.8531	0.8531	0.8531	0.8531
bl14	0.8531	0.8531	0.8611	0.8611	0.8611	0.8611
bl20	0.8531	0.8531	0.8531	0.8531	0.8531	0.8531

Table S4. Original data, AUC, wavelet smoothing method, logarithm transformation

Method	Accuracy	AUC
Linear spline	0.5500	0.6081
Cubic spline	0.5500	0.6081
Quintic spline	0.5500	0.6081
Fourier 20 basis	0.6200	0.6996
Fourier 40 basis	0.6333	0.7436
Fourier 60 basis	0.6533	0.6872

Table S5. Original data, spline and Fourier smoothing methods, angular velocity transformation

DLs	1	2	3	4	5	6
haar	0.5733	0.5933	0.5933	0.6133	0.6300	0.5933
d4	0.6833	0.6333	0.6733	0.6333	0.6900	0.6900
d6	0.6500	0.7200	0.7400	0.7033	0.7400	0.7233
d8	0.6867	0.6600	0.7233	0.6867	0.6500	0.6867
d16	0.7100	0.7500	0.7333	0.7467	0.7667	0.7667
la8	0.7033	0.7100	0.6733	0.6733	0.6733	0.6733
la16	0.7067	0.6733	0.6500	0.6667	0.6667	0.6667
la20	0.7067	0.6700	0.6867	0.6500	0.6833	0.6833
bl14	0.6900	0.7233	0.6900	0.7267	0.7233	0.7433
bl20	0.7067	0.6700	0.6900	0.6700	0.6867	0.6867

Table S6. Original data, accuracy, wavelet smoothing method, angular velocity transformation

DLs	1	2	3	4	5	6
haar	0.6026	0.6737	0.6684	0.6626	0.6796	0.6604
d4	0.6962	0.7423	0.7130	0.7236	0.7293	0.7213
d6	0.6928	0.7103	0.7134	0.7091	0.7159	0.7020
d8	0.7079	0.6523	0.7291	0.6869	0.6737	0.7060
d16	0.7300	0.7549	0.7799	0.7743	0.7783	0.7783
la8	0.7334	0.7217	0.7177	0.7238	0.7198	0.7158
la16	0.7467	0.7333	0.6730	0.7180	0.7100	0.7100
la20	0.7467	0.7053	0.6980	0.6573	0.6768	0.6768
bl14	0.7244	0.7630	0.7488	0.7660	0.7599	0.7679
bl20	0.7467	0.7164	0.6918	0.6989	0.6937	0.6857

Table S7. Original data, AUC, wavelet smoothing method, angular velocity transformation

Method	Accuracy	AUC
Linear spline	0.4500	0.6554
Cubic spline	0.4667	0.6610
Quintic spline	0.4667	0.6610
Fourier 20 basis	0.5033	0.6194
Fourier 40 basis	0.5033	0.6302
Fourier 60 basis	0.5067	0.6283

Table S8. First noisy data set, spline and Fourier smoothing methods, logarithm transformation

DLs	1	2	3	4	5	6
haar	0.4833	0.4833	0.4833	0.4833	0.4667	0.4667
d4	0.4500	0.4700	0.4667	0.4867	0.4900	0.4900
d6	0.4500	0.4667	0.4667	0.5433	0.5067	0.5067
d8	0.4667	0.4833	0.4667	0.4867	0.4867	0.4667
d16	0.4500	0.5000	0.5000	0.5033	0.4867	0.5033
la8	0.4500	0.4833	0.4833	0.5067	0.4700	0.4500
la16	0.4500	0.4667	0.4867	0.4867	0.4867	0.4700
la20	0.4500	0.4667	0.4667	0.4867	0.4867	0.4700
bl14	0.4500	0.4667	0.4500	0.5067	0.5067	0.4867
bl20	0.4500	0.4833	0.4667	0.4667	0.4667	0.4667

Table S9. First noisy data set, accuracy, wavelet smoothing method, logarithm transformation

DLs	1	2	3	4	5	6
haar	0.6527	0.6391	0.6394	0.6434	0.6351	0.6462
d4	0.6391	0.6594	0.6613	0.6754	0.6958	0.6958
d6	0.6391	0.6530	0.6530	0.6527	0.6148	0.6228
d8	0.6530	0.6647	0.6511	0.6471	0.6163	0.6363
d16	0.6471	0.6650	0.6650	0.6191	0.6163	0.6191
la8	0.6471	0.6527	0.6567	0.6431	0.6148	0.6348
la16	0.6471	0.6471	0.6271	0.6160	0.6191	0.6274
la20	0.6471	0.6530	0.6391	0.6191	0.6191	0.6274
bl14	0.6471	0.6650	0.6594	0.6431	0.6320	0.6231
bl20	0.6471	0.6567	0.6471	0.6511	0.6511	0.6511

Table S10. First noisy data set, AUC, wavelet smoothing method, logarithm transformation

Method	Accuracy	AUC
Linear spline	0.3900	0.6503
Cubic spline	0.3900	0.6503
Quintic spline	0.3900	0.6503
Fourier 20 basis	0.3933	0.6649
Fourier 40 basis	0.4167	0.6797
Fourier 60 basis	0.4467	0.6639

Table S11. First noisy data set, spline and Fourier smoothing methods, angular velocity transformation

DLs	1	2	3	4	5	6
haar	0.3933	0.4133	0.3967	0.3900	0.4267	0.3767
d4	0.4333	0.4667	0.4333	0.3933	0.3867	0.3700
d6	0.4133	0.4333	0.3767	0.4700	0.3567	0.3933
d8	0.4700	0.4133	0.4500	0.4367	0.3567	0.3767
d16	0.3933	0.4333	0.4133	0.4700	0.3400	0.3767
la8	0.4133	0.4333	0.4300	0.3733	0.3367	0.3533
la16	0.3933	0.4133	0.4300	0.3933	0.3733	0.3700
la20	0.3933	0.4167	0.4133	0.3733	0.3900	0.3900
bl14	0.4333	0.4333	0.4500	0.4133	0.3567	0.3567
bl20	0.3933	0.4167	0.4300	0.3933	0.4100	0.4100

Table S12. First noisy data set, accuracy, wavelet smoothing method, angular velocity transformation

DLs	1	2	3	4	5	6
haar	0.7000	0.6689	0.6677	0.6288	0.6001	0.6566
d4	0.6920	0.6439	0.6470	0.6658	0.6269	0.6448
d6	0.7040	0.6861	0.6341	0.6288	0.6541	0.6347
d8	0.6988	0.6624	0.6183	0.6581	0.6686	0.6486
d16	0.7434	0.6790	0.6246	0.6608	0.6664	0.6366
la8	0.7172	0.6538	0.6451	0.6482	0.6686	0.6587
la16	0.7372	0.6538	0.6347	0.6427	0.6427	0.6408
la20	0.7400	0.6969	0.6110	0.6396	0.6288	0.6288
bl14	0.6760	0.6778	0.6131	0.6562	0.6646	0.6646
bl20	0.7400	0.6981	0.6091	0.6276	0.6220	0.6180

Table S13. First noisy data set, AUC, wavelet smoothing method, angular velocity transformation

Method	Accuracy	AUC
Linear spline	0.4633	0.5872
Cubic spline	0.4633	0.5709
Quintic spline	0.4633	0.5829
Fourier 20 basis	0.4800	0.5603
Fourier 40 basis	0.5000	0.5807
Fourier 60 basis	0.5000	0.5807

Table S14. Second noisy data set, spline and Fourier smoothing methods, logarithm transformation

DLs	1	2	3	4	5	6
haar	0.4633	0.4633	0.4800	0.4800	0.5000	0.5000
d4	0.4633	0.4633	0.4800	0.4967	0.4967	0.4967
d6	0.4633	0.4800	0.4633	0.4800	0.4800	0.4800
d8	0.4633	0.4633	0.4633	0.4800	0.4800	0.4800
d16	0.4633	0.4633	0.4633	0.4800	0.4800	0.4800
la8	0.4633	0.4633	0.4633	0.4633	0.4800	0.4800
la16	0.4633	0.4633	0.4633	0.4633	0.4633	0.4633
la20	0.4633	0.4633	0.4800	0.4800	0.4800	0.4800
bl14	0.4633	0.4633	0.4633	0.4800	0.4800	0.4800
bl20	0.4633	0.4633	0.4633	0.4800	0.4800	0.4800

Table S15. Second noisy data set, accuracy, wavelet smoothing method, logarithm transformation

DLs	1	2	3	4	5	6
haar	0.5952	0.5952	0.5761	0.5841	0.6001	0.6001
d4	0.5832	0.5848	0.5681	0.5634	0.5634	0.5634
d6	0.5832	0.5681	0.5832	0.5758	0.5773	0.5718
d8	0.5912	0.5789	0.5653	0.5662	0.5678	0.5622
d16	0.5832	0.5709	0.5709	0.5718	0.5718	0.5718
la8	0.5832	0.5829	0.5789	0.5909	0.5798	0.5798
la16	0.5832	0.5789	0.5789	0.5829	0.5829	0.5829
la20	0.5832	0.5792	0.5801	0.5718	0.5718	0.5718
bl14	0.5832	0.5792	0.5829	0.5718	0.5718	0.5718
bl20	0.5832	0.5709	0.5909	0.5798	0.5718	0.5718

Table S16. Second noisy data set, AUC, wavelet smoothing method, logarithm transformation

Method	Accuracy	AUC
Linear spline	0.4400	0.6112
Cubic spline	0.4600	0.5937
Quintic spline	0.4400	0.6451
Fourier 20 basis	0.5800	0.6514
Fourier 40 basis	0.5600	0.6598
Fourier 60 basis	0.5833	0.6798

Table S17. Second noisy data set, spline and Fourier smoothing methods, angular velocity transformation

DLs	1	2	3	4	5	6
haar	0.4600	0.4400	0.4400	0.4233	0.4600	0.4600
d4	0.4800	0.4400	0.4600	0.4200	0.4400	0.4400
d6	0.4400	0.4233	0.4400	0.4400	0.4400	0.4400
d8	0.4600	0.4600	0.4400	0.4200	0.4400	0.4600
d16	0.4600	0.4400	0.4400	0.4600	0.4400	0.4600
la8	0.4600	0.4600	0.4600	0.4600	0.4400	0.4600
la16	0.4600	0.4600	0.4400	0.4433	0.4600	0.4600
la20	0.4600	0.4400	0.4400	0.4433	0.4600	0.4600
bl14	0.4600	0.4400	0.4400	0.4400	0.4400	0.4400
bl20	0.4600	0.4400	0.4400	0.4433	0.4600	0.4600

Table S18. Second noisy data set, accuracy, wavelet smoothing method, angular velocity transformation

DLs	1	2	3	4	5	6
haar	0.6112	0.6112	0.6112	0.6331	0.6239	0.6239
d4	0.6232	0.6152	0.5992	0.6232	0.6116	0.6239
d6	0.6484	0.6442	0.6152	0.6236	0.5912	0.6017
d8	0.6340	0.6223	0.6112	0.6232	0.6079	0.6112
d16	0.6272	0.6303	0.6112	0.6112	0.5912	0.6072
la8	0.6152	0.6143	0.5952	0.6076	0.6116	0.6156
la16	0.6236	0.6143	0.6112	0.6374	0.6112	0.6152
la20	0.6236	0.6383	0.6112	0.6371	0.6112	0.6152
bl14	0.6281	0.6423	0.6112	0.6152	0.5992	0.6236
bl20	0.6236	0.6383	0.6112	0.6211	0.6143	0.6060

Table S19. Second noisy data set, AUC, wavelet smoothing method, angular velocity transformation

Method	Accuracy	AUC
Linear spline	0.5267	0.6233
Cubic spline	0.5067	0.6273
Quintic spline	0.5067	0.6329
Fourier 20 basis	0.4433	0.6797
Fourier 40 basis	0.4867	0.6101
Fourier 60 basis	0.4867	0.6277

Table S20. Third noisy data set, spline and Fourier smoothing methods, logarithm transformation

DLs	1	2	3	4	5	6
haar	0.4867	0.4867	0.4833	0.4867	0.4667	0.4667
d4	0.4867	0.4633	0.4667	0.4800	0.5033	0.5233
d6	0.4333	0.4067	0.4667	0.4833	0.4667	0.4667
d8	0.4500	0.4067	0.3900	0.4467	0.4500	0.4467
d16	0.4867	0.4267	0.4467	0.4867	0.4667	0.4267
la8	0.4700	0.4867	0.4267	0.4833	0.4667	0.4633
la16	0.4700	0.4467	0.4467	0.4067	0.4267	0.4267
la20	0.4700	0.4467	0.4467	0.4067	0.4267	0.4267
bl14	0.4500	0.4067	0.4467	0.4433	0.4300	0.4633
bl20	0.4700	0.4467	0.4267	0.4267	0.4667	0.4467

Table S21. Third noisy data set, accuracy, wavelet smoothing method, logarithm transformation

DLs	1	2	3	4	5	6
haar	0.6609	0.6449	0.6911	0.6918	0.6782	0.6782
d4	0.6609	0.6449	0.6911	0.6918	0.6782	0.6782
d6	0.6344	0.6833	0.6286	0.6424	0.6286	0.6341
d8	0.6443	0.6698	0.6917	0.6767	0.6388	0.6298
d16	0.6692	0.6757	0.6446	0.6548	0.6421	0.6757
la8	0.6553	0.6073	0.6406	0.6184	0.6046	0.6464
la16	0.6553	0.6353	0.6437	0.6917	0.6677	0.6597
la20	0.6553	0.6517	0.6397	0.6781	0.6652	0.6597
bl14	0.6273	0.6781	0.6557	0.6760	0.6624	0.6560
bl20	0.6473	0.6637	0.6621	0.6661	0.6301	0.6381

Table S22. Third noisy data set, AUC, wavelet smoothing method, logarithm transformation

Method	Accuracy	AUC
Linear spline	0.4133	0.6877
Cubic spline	0.4133	0.6877
Quintic spline	0.4133	0.6877
Fourier 20 basis	0.4700	0.6541
Fourier 40 basis	0.4667	0.6350
Fourier 60 basis	0.4833	0.6271

Table S23. Third noisy data set, spline and Fourier smoothing methods, angular velocity transformation

DLs	1	2	3	4	5	6
haar	0.4500	0.5067	0.4900	0.4500	0.4500	0.4300
d4	0.3900	0.4900	0.4900	0.4500	0.4533	0.4300
d6	0.4233	0.4900	0.4700	0.4500	0.4333	0.4500
d8	0.4033	0.5233	0.4300	0.4500	0.4900	0.4700
d16	0.4100	0.5067	0.4500	0.4300	0.4900	0.4533
la8	0.4133	0.5267	0.5100	0.4300	0.4533	0.4300
la16	0.3733	0.5267	0.4333	0.4300	0.4700	0.4133
la20	0.3900	0.4900	0.4333	0.4300	0.4700	0.4133
bl14	0.4233	0.5067	0.4900	0.4300	0.4533	0.4333
bl20	0.4100	0.4900	0.4533	0.4300	0.4700	0.4300

Table S24. Third noisy data set, accuracy, wavelet smoothing method, angular velocity transformation

DLs	1	2	3	4	5	6
haar	0.6359	0.6562	0.6489	0.6600	0.6356	0.6821
d4	0.6519	0.6606	0.6282	0.6646	0.6772	0.6738
d6	0.6601	0.6793	0.6646	0.6664	0.6852	0.6710
d8	0.6444	0.6352	0.6628	0.6828	0.6882	0.6550
d16	0.6821	0.6288	0.6788	0.6988	0.6726	0.6522
la8	0.6719	0.6728	0.6694	0.6566	0.6856	0.6793
la16	0.6870	0.6728	0.6723	0.6704	0.6661	0.6682
la20	0.6811	0.6494	0.6624	0.6788	0.6661	0.6682
bl14	0.6872	0.6189	0.6534	0.6984	0.6692	0.6738
bl20	0.6691	0.6618	0.6541	0.6948	0.6824	0.6877

Table S25. Third noisy data set, AUC, wavelet smoothing method, angular velocity transformation

Method	Accuracy	AUC
Linear spline	0.6600	0.6649
Cubic spline	0.6600	0.6788
Quintic spline	0.6767	0.6696
Fourier 20 basis	0.6633	0.6557
Fourier 40 basis	0.6600	0.6942
Fourier 60 basis	0.6467	0.6393

Table S26. Fourth noisy data set, spline and Fourier smoothing methods, logarithm transformation

DLs	1	2	3	4	5	6
haar	0.6200	0.6500	0.6467	0.5700	0.6300	0.5700
d4	0.7200	0.6600	0.5933	0.6133	0.6100	0.6633
d6	0.7067	0.6467	0.5567	0.6133	0.6333	0.6333
d8	0.6633	0.5900	0.6100	0.6467	0.6433	0.6467
d16	0.6833	0.7000	0.5900	0.6100	0.5900	0.6300
la8	0.7067	0.6233	0.6433	0.6467	0.6867	0.6833
la16	0.6867	0.6600	0.6267	0.6633	0.6267	0.6467
la20	0.6867	0.6800	0.5700	0.6633	0.6300	0.6800
bl14	0.7067	0.5900	0.6033	0.6867	0.6267	0.6100
bl20	0.6867	0.6800	0.5700	0.6300	0.6167	0.6833

Table S27. Fourth noisy data set, accuracy, wavelet smoothing method, logarithm transformation

DLs	1	2	3	4	5	6
haar	0.6409	0.6784	0.6270	0.5860	0.6183	0.6196
d4	0.7136	0.6642	0.6057	0.6236	0.6319	0.7046
d6	0.6748	0.6544	0.5928	0.6564	0.6574	0.6263
d8	0.6612	0.6800	0.6831	0.6963	0.6791	0.6557
d16	0.6720	0.6600	0.6390	0.5924	0.5894	0.6467
la8	0.6957	0.6909	0.6989	0.7416	0.7096	0.7007
la16	0.6704	0.7001	0.6994	0.7053	0.6788	0.6807
la20	0.6649	0.6593	0.6329	0.6643	0.6313	0.6638
bl14	0.6788	0.6553	0.6853	0.6824	0.6803	0.6856
bl20	0.6704	0.6687	0.5910	0.6366	0.6538	0.6498

Table S28. Fourth noisy data set, AUC, wavelet smoothing method, logarithm transformation

Method	Accuracy	AUC
Linear spline	0.4433	0.6534
Cubic spline	0.4433	0.6534
Quintic spline	0.4433	0.6534
Fourier 20 basis	0.4833	0.6408
Fourier 40 basis	0.5433	0.5954
Fourier 60 basis	0.4800	0.6161

Table S29. Fourth noisy data set, spline and Fourier smoothing methods, angular velocity transformation

DLs	1	2	3	4	5	6
haar	0.4633	0.5433	0.5400	0.4267	0.4633	0.4400
d4	0.5267	0.4833	0.4600	0.3900	0.4800	0.4233
d6	0.4867	0.4500	0.4667	0.4300	0.4667	0.4267
d8	0.3733	0.4667	0.4467	0.4300	0.4267	0.4267
d16	0.4833	0.5100	0.4633	0.4500	0.4467	0.4433
la8	0.4667	0.4867	0.4267	0.3700	0.4633	0.4233
la16	0.4467	0.4833	0.4633	0.4833	0.4833	0.4267
la20	0.4467	0.5600	0.4667	0.5000	0.5033	0.4267
bl14	0.4867	0.5100	0.4267	0.4100	0.4667	0.4433
bl20	0.4667	0.5233	0.4667	0.4500	0.4667	0.4267

Table S30. Fourth noisy data set, accuracy, wavelet smoothing method, angular velocity transformation

DLs	1	2	3	4	5	6
haar	0.6490	0.6062	0.5883	0.6276	0.6174	0.6131
d4	0.6512	0.5980	0.6036	0.6190	0.6088	0.6310
d6	0.6540	0.6084	0.6220	0.6477	0.6494	0.6273
d8	0.6652	0.5711	0.6053	0.6267	0.6559	0.6350
d16	0.6493	0.5798	0.6060	0.6331	0.6383	0.6211
la8	0.6426	0.5456	0.5927	0.6402	0.6116	0.6310
la16	0.6266	0.5902	0.5986	0.6211	0.6316	0.6713
la20	0.5881	0.5617	0.6078	0.6128	0.6276	0.6713
bl14	0.6368	0.6063	0.6069	0.6621	0.6310	0.6378
bl20	0.6041	0.5796	0.6238	0.6522	0.6298	0.6713

Table S31. Fourth noisy data set, AUC, wavelet smoothing method, angular velocity transformation

Method	Accuracy	AUC
Linear spline	0.4400	0.6451
Cubic spline	0.4400	0.6451
Quintic spline	0.4400	0.6451
Fourier 20 basis	0.4033	0.6957
Fourier 40 basis	0.4367	0.6609
Fourier 60 basis	0.4733	0.6763

Table S32. Fifth noisy data set, spline and Fourier smoothing methods, logarithm transformation

DLs	1	2	3	4	5	6
haar	0.4600	0.4200	0.4400	0.4400	0.4600	0.4600
d4	0.4233	0.4267	0.4267	0.4233	0.4433	0.4433
d6	0.4267	0.4267	0.4800	0.4433	0.4267	0.4067
d8	0.4600	0.4833	0.4800	0.4400	0.4200	0.4600
d16	0.4400	0.4667	0.4400	0.4400	0.4733	0.4733
la8	0.4233	0.4233	0.4233	0.4433	0.4233	0.4400
la16	0.4433	0.4800	0.4800	0.4433	0.4233	0.4033
la20	0.4433	0.4667	0.5000	0.4433	0.4400	0.4400
bl14	0.4267	0.4467	0.4600	0.4400	0.4233	0.4067
bl20	0.4267	0.4667	0.4600	0.4433	0.4233	0.4433

Table S33. Fifth noisy data set, accuracy, wavelet smoothing method, logarithm transformation

DLs	1	2	3	4	5	6
haar	0.6291	0.6778	0.6852	0.6602	0.6263	0.6343
d4	0.6741	0.6276	0.6534	0.6611	0.6451	0.6451
d6	0.6494	0.6454	0.6411	0.6710	0.6543	0.6703
d8	0.6411	0.6341	0.6541	0.6821	0.6981	0.6741
d16	0.6698	0.6063	0.6901	0.6976	0.6849	0.7163
la8	0.6741	0.6491	0.6571	0.6451	0.6540	0.6651
la16	0.6541	0.6402	0.6458	0.6646	0.6734	0.6894
la20	0.6621	0.6094	0.6322	0.6204	0.6571	0.6571
bl14	0.6494	0.6334	0.6538	0.6618	0.6590	0.6503
bl20	0.6454	0.6094	0.6402	0.6454	0.6654	0.6124

Table S34. Fifth noisy data set, AUC, wavelet smoothing method, logarithm transformation

Method	Accuracy	AUC
Linear spline	0.4367	0.5764
Cubic spline	0.4367	0.5764
Quintic spline	0.4367	0.5764
Fourier 20 basis	0.4367	0.5933
Fourier 40 basis	0.4800	0.5884
Fourier 60 basis	0.4967	0.5203

Table S35. Fifth noisy data set, spline and Fourier smoothing methods, angular velocity transformation

DLs	1	2	3	4	5	6
haar	0.4567	0.4567	0.4533	0.4200	0.4533	0.4367
d4	0.4733	0.4900	0.4733	0.4733	0.4533	0.4200
d6	0.4400	0.4933	0.4733	0.4733	0.4367	0.4200
d8	0.4967	0.4400	0.4733	0.4533	0.4367	0.4367
d16	0.4533	0.4800	0.4733	0.4733	0.4567	0.4200
la8	0.4933	0.4800	0.4167	0.4567	0.4533	0.4367
la16	0.4933	0.4967	0.4533	0.4167	0.4733	0.4367
la20	0.4933	0.4767	0.4367	0.4367	0.4733	0.4367
bl14	0.4400	0.4967	0.4167	0.4733	0.4533	0.4200
bl20	0.4933	0.4767	0.4367	0.4733	0.4533	0.4533

Table S36. Fifth noisy data set, accuracy, wavelet smoothing method, angular velocity transformation

DLs	1	2	3	4	5	6
haar	0.5927	0.5912	0.5942	0.5751	0.5829	0.5678
d4	0.5718	0.5909	0.5940	0.5940	0.6032	0.5653
d6	0.5927	0.5989	0.5952	0.5940	0.6048	0.5733
d8	0.6059	0.5982	0.5980	0.5721	0.5817	0.5844
d16	0.5906	0.5514	0.5952	0.5601	0.5804	0.5706
la8	0.5834	0.5742	0.6213	0.5968	0.6032	0.5737
la16	0.5918	0.5878	0.6112	0.6093	0.5817	0.5737
la20	0.5918	0.5579	0.5973	0.5893	0.5817	0.5737
bl14	0.5902	0.5798	0.6213	0.5940	0.5937	0.5733
bl20	0.5918	0.5619	0.5973	0.5601	0.6029	0.5681

Table S37. Fifth noisy data set, AUC, wavelet smoothing method, angular velocity transformation

1.3 Logistic regression results

The ANOVA tables for the two linear models and the regression results for AUC are presented in this section.

Variables	Df	Sum of Sq	Mean Sq	F value	p value	
Transformation	1	28.92	28.921	201.3827	$< 2\,10^{-16}$	***
Smoothing method	2	0.20	0.101	0.7023	0.4955	
Noise variance (α)	1	81.94	81.941	570.5719	$< 2\,10^{-16}$	***
Noise autocorrelation (β)	1	5.50	5.501	8.3024	$6.36\,10^{-10}$	***
Residuals	7914	1136.55	0.144			

Table S38. ANOVA table for linear model with accuracy target variable.

	Res Df	RSS	Df	Sum of Sq	F value	p value	
Null model	7919	1253.1					
Final model	7914	1136.5	5	116.56	162.33	$< 2\,10^{-16}$	***

Table S39. ANOVA table for linear model with accuracy target variable.

Variables	Coefficients	stddev	CI	
Intercept	0.676	0.015	(0.645,0.707)	*
Transformation logarithm	0.052	0.007	(0.038,0.064)	*
Smoothing method spline	0.002	0.021	(-0.035,0.043)	
Smoothing method wavelet	0.004	0.015	(-0.027,0.033)	
Noise variance (α)	-1.564	0.061	(-1.679,-1.433)	*
Noise autocorrelation (β)	-1.838	0.065	(-1.969,-1.714)	*

Table S40. Linear regression model for AUC target variable with a bootstrap procedure.

Variables	Df	Sum of Sq	Mean Sq	F value	p value	
Transformation	1	5.31	5.312	70.2698	$< 2\,10^{-16}$	***
Smoothing method	2	0.01	0.003	0.0406	0.9602	
Noise variance (α)	1	18.69	18.689	247.2439	$< 2\,10^{-16}$	***
Noise autocorrelation (β)	1	32.83	32.829	434.3070	$< 2\,10^{-16}$	***
Residuals	7914	501.55	0.063			

Table S41. ANOVA table for linear model with AUC target variable.

	Res Df	RSS	Df	Sum of Sq	F value	p value	
Null model	7919	655.06					
Final model	7914	598.22	5	56.837	150.38	$< 2\,10^{-16}$	***

Table S42. ANOVA table for linear model with AUC target variable.

1.4 Detailed results of classification task on human behaviour study dataset

Method	Accuracy	AUC
Linear spline	0.5875	0.6582
Cubic spline	0.6000	0.6727
Quintic spline	0.6000	0.6727
Fourier 20 basis	0.6000	0.6278
Fourier 40 basis	0.6125	0.6436
Fourier 60 basis	0.6125	0.6419

Table S43. Real data set, spline and Fourier smoothing methods, logarithm transformation

DLs	1	2	3	4	5	6
haar	0.6000	0.6000	0.6000	0.6000	0.6000	0.6000
d4	0.6000	0.6000	0.6000	0.6000	0.6000	0.6000
d6	0.6000	0.6000	0.6000	0.6000	0.6000	0.6000
d8	0.6000	0.6000	0.6000	0.6000	0.6000	0.6000
d16	0.6000	0.6000	0.6000	0.6000	0.6000	0.6000
la8	0.6000	0.6000	0.6000	0.6000	0.6000	0.6000
la16	0.6000	0.6000	0.6000	0.6000	0.6000	0.6000
la20	0.6000	0.6000	0.6000	0.6000	0.6000	0.6000
bl14	0.6000	0.6000	0.6000	0.6000	0.6000	0.6000
bl20	0.6000	0.6000	0.6000	0.6000	0.6000	0.6000

Table S44. Real data set, accuracy, wavelet smoothing method, logarithm transformation

DLs	1	2	3	4	5	6
haar	0.6646	0.6646	0.6646	0.6646	0.6646	0.6646
d4	0.6646	0.6646	0.6646	0.6646	0.6646	0.6646
d6	0.6646	0.6646	0.6646	0.6646	0.6646	0.6646
d8	0.6646	0.6646	0.6646	0.6646	0.6646	0.6646
d16	0.6646	0.6646	0.6646	0.6646	0.6646	0.6646
la8	0.6646	0.6646	0.6646	0.6646	0.6646	0.6646
la16	0.6646	0.6646	0.6646	0.6646	0.6646	0.6646
la20	0.6646	0.6646	0.6646	0.6646	0.6646	0.6646
bl14	0.6646	0.6646	0.6646	0.6646	0.6646	0.6646
bl20	0.6646	0.6646	0.6646	0.6646	0.6646	0.6646

Table S45. Real data set, AUC, wavelet smoothing method, logarithm transformation

Method	Accuracy	AUC
Linear spline	0.5875	0.6002
Cubic spline	0.5875	0.6002
Quintic spline	0.5875	0.6002
Fourier 20 basis	0.6125	0.6572
Fourier 40 basis	0.6250	0.6588
Fourier 60 basis	0.6250	0.6588

Table S46. Real data set, spline and Fourier smoothing methods, angular velocity transformation

DLs	1	2	3	4	5	6
haar	0.6250	0.6250	0.6250	0.6250	0.6250	0.6250
d4	0.6250	0.6250	0.6250	0.6250	0.6250	0.6250
d6	0.6250	0.6250	0.6250	0.6250	0.6250	0.6250
d8	0.6250	0.6250	0.6250	0.6250	0.6250	0.6250
d16	0.6250	0.6250	0.6250	0.6250	0.6250	0.6250
la8	0.6250	0.6250	0.6250	0.6250	0.6250	0.6250
la16	0.6250	0.6250	0.6250	0.6250	0.6250	0.6250
la20	0.6250	0.6250	0.6250	0.6250	0.6250	0.6250
bl14	0.6250	0.6250	0.6250	0.6250	0.6250	0.6250
bl20	0.6250	0.6250	0.6250	0.6250	0.6250	0.6250

Table S47. Real data set, accuracy, wavelet smoothing method, angular velocity transformation

DLs	1	2	3	4	5	6
haar	0.5714	0.5714	0.5714	0.5714	0.5714	0.5714
d4	0.5714	0.5714	0.5714	0.5714	0.5714	0.5714
d6	0.5714	0.5714	0.5714	0.5714	0.5714	0.5714
d8	0.5714	0.5714	0.5714	0.5714	0.5714	0.5714
d16	0.5714	0.5714	0.5714	0.5714	0.5714	0.5714
la8	0.5714	0.5714	0.5714	0.5714	0.5714	0.5714
la16	0.5714	0.5714	0.5714	0.5714	0.5714	0.5714
la20	0.5714	0.5714	0.5714	0.5714	0.5714	0.5714
bl14	0.5714	0.5714	0.5714	0.5714	0.5714	0.5714
bl20	0.5714	0.5714	0.5714	0.5714	0.5714	0.5714

Table S48. Real data set, AUC, wavelet smoothing method, angular velocity transformation

2 Introduction to quaternion theory

Some useful notion about quaternion theory are reported to make easier the understanding to the reader.

Quaternion algebra has been fully studied and applied since the development of computer graphics, specifically to approach the problem of the motion of a rigid body.
An extensive introduction on quaternion algebra can be found in[1]. Some information useful to the comprehension of the paper are reported in the following paragraph.
Quaternions were first defined by Hamilton in 1843, as a generalization of the complex numbers, with three imaginary units: **i, j, k**.
Each quaternion can be represented as $q = w + x\mathbf{i} + y\mathbf{j} + z\mathbf{k}$ where w, x, y, and z are real numbers: w is referred to as the real part of q and x, y, and z are the imaginary parts.
Imaginary units satisfy the conditions

$$\mathbf{i}^2 = \mathbf{j}^2 = \mathbf{k}^2 = \mathbf{ijk} = -1$$

This implies that quaternion multiplication is not commutative. Quaternions are often represented as the 4-dimensional vector of their real components: $q = (w, x, y, z)$.
The norm of a quaternion is defined to be $|q| = \sqrt{w^2 + x^2 + y^2 + z^2}$.
These different types of quaternions and operations on quaternions include:

- The pure quaternion $q = (0, x, y, z)$.
- The identity quaternion $q = (1, 0, 0, 0)$.
- The unit quaternion $q = (w, x, y, z)$, where $|q| = 1$.
- The conjugate quaternion $\bar{q} = (w, -x, -y, -z)$.

- The quaternion inverse $q^{-1} = \bar{q}/|q|$
  If we define $\mathbf{v} = (x, y, z) \in \mathbb{R}^3$:
- The logarithm $\log(q) = (\log(|q|), \frac{x}{|\mathbf{v}|} \arccos(\frac{w}{|q|}), \frac{y}{|\mathbf{v}|} \arccos(\frac{w}{|q|}), \frac{z}{|\mathbf{v}|} \arccos(\frac{w}{|q|}))$,
- The exponential $\exp(q) = \exp(w)(\cos(|\mathbf{v}|), \frac{x}{|\mathbf{v}|} \sin(|\mathbf{v}|), \frac{y}{|\mathbf{v}|} \sin(|\mathbf{v}|), \frac{z}{|\mathbf{v}|} \sin(|\mathbf{v}|))$.

Unit quaternions are specifically used for describing rotations in three dimensions. Their intrinsic properties confer on them a number of advantages over the other classical representations, such as Euler angles and rotation matrices (see[1]).
A unit quaternion (i.e. a finite rotation) can also be represented as a single rotation about an appropriately chosen axis. So the quaternion can also be defined as an angle $\theta \in \mathbb{R}$ and a three element vector $\mathbf{v} = (v_x, v_y, v_z) \in \mathbb{S}^2$, where $\mathbb{S}^2 := \{\mathbf{v} \in \mathbb{R}^3 : \|\mathbf{v}\| = 1\}$:

$$q = q(\theta, \mathbf{v}) = (\cos\frac{\theta}{2}, v_x \sin\frac{\theta}{2}, v_y \sin\frac{\theta}{2}, v_z \sin\frac{\theta}{2})$$

The inverse mapping is defined by the equations:

$$\begin{cases} \theta(q) := 2\arccos(w) \\ \mathbf{v}(q) = \frac{(x,y,z)}{|(x,y,z)|} = \frac{(x,y,z)}{\sqrt{1-w^2}} \end{cases} \tag{1}$$

We remark that the map from the unit quaternions to the rotations is not injective: for every rotation, two quaternions, +q and –q, lying at antipodal points of a hypersphere, correspond to it.
For unit quaternions, the exponential and logarithm maps assume specific formulations and meanings. In fact, Euler's identity for complex numbers generalizes to quaternions, i.e. $\exp(\mathbf{v}\theta) = \cos\theta + \mathbf{v}\sin\theta$, results derived from the power series representation for $\exp(x)$. From this formulation is also possible to define the logarithm of a unit quaternion, $\log(q) = \mathbf{v}\theta \in \mathbb{R}^3$. It is important to note that the noncommutativity of quaternion multiplication invalidates the standard identities for the exponential and logarithm functions, as described in[2].
Let us suppose that $\mathbf{r} = (r_x, r_y, r_z)$ is a point in 3D space and q_r represents the same vector in quaternionic form, $q_r = (0, r_x, r_y, r_z)$. The vector q'_r, resulting from the rotation by an angle θ around the axis $\mathbf{v}$, can be calculated by quaternion multiplication as $q'_r = q q_r q^{-1}$
Let $q_1 = (w_1, x_1, y_1, z_1)$ and $q_2 = (w_2, x_2, y_2, z_2)$ be unit quaternions. The distance between them is the geodesic distance defined as follows:

$$d(q_1, q_2) = 2\arccos(|q_1 \cdot q_2|) = 2\arccos(w_1w_2 + x_1x_2 + y_1y_2 + z_1z_2). \tag{2}$$

This definition is functionally equivalent to the geodesic distance on the unit sphere, defined by $d(q_1, q_2) = \|log(q_1 q_2^T)\|$. The average of n quaternions $q_1, ..., q_n$ is defined as the sample Fréchet mean:

$$\bar{q} = avg(q_i) = argmin_q(\sum_{i=1}^{n} d^2(q, q_i))$$

Quaternion time series

A quaternion time series is a sequence of (unit) quaternions $q^{(i)}$, $i \in \{1, ..., n\}$.
One of the distances between two quaternion time series $\mathbf{q_1}$ and $\mathbf{q_2}$ is defined as the l^2 geodesic distance:

$$d(\mathbf{q_1}, \mathbf{q_2}) = \sqrt{\sum_{i=1}^{n} d^2(q_1^{(i)}, q_2^{(i)})} =$$
$$= \sqrt{\sum_{i=1}^{n} (\arccos(w_1^{(i)} w_2^{(i)} + x_1^{(i)} x_2^{(i)} + y_1^{(i)} y_2^{(i)} + z_1^{(i)} z_2^{(i)}))^2}$$

A more elastic distance measure defined in[3] is called Dynamic Time Warping (DTW).

$$DTW(\mathbf{q}_1, \mathbf{q}_2) = \min_{\omega} d_{\omega}(\mathbf{q}_1, \mathbf{q}_2) = \min_{\omega} \sum_{i=1}^{T} d(q_1^{(\omega(i))}, q_2^{(\omega(i))}) \tag{3}$$

where $\omega(i)$ is the warping path, i.e. a function which defines a mapping between index i to index j. The distances implemented in Equation 3 can be chosen according to the purpose, but the geodesic distance is the most common choice.
In the context of quaternion time series, quaternion spherical linear interpolation (SLERP) is an extension of linear interpolation along a plane to spherical interpolation in three dimensions, as first proposed in[4]. Given two quaternions, q_1 and q_2, SLERP interpolates a new quaternion, q_0, along the great circle that connects q_1 and q_2:

$$q_0 = \frac{\sin((1-T)\theta)}{\sin(\theta)} q_1 + \frac{\sin(T\theta)}{\sin(\theta)} q_2$$

where T is the interpolation coefficient that determines how close the new quaternion is to either q_1 and q_2, and θ is one-half the angular distance between q_1 and q_2.
The spherical quadrangle interpolation (SQUAD) is a spline-based interpolation of rotations (unit quaternion), also known as spherical cubic interpolation.
If $\{q_i\}_{i=1,\dots N}$ is a sequence of N quaternions, then define an 'helper' quaternion $s_i = \exp(-\frac{log(q_{i+1}q_i^{-1})+log(q_{i-1}q_i^{-1})}{4})q_i$.
Then the interpolation is given by

$$squad(q_i, q_{i+1}, s_i, s_{i+1}, T) = slerp(slerp(q_i, q_{i+1}, T), slerp(s_i, s_{i+1}, T), 2T(1-T))$$

where q_i, q_{i+1} represent the start and destination rotations and T is the interpolation parameter, which lies in the interval $[0,1]$.
Another crucial point in our work is the definition of angular velocity for a quaternion time series. A unit quaternion time series is the discrete representation of a curve $q: T \to \mathbb{H}_1$. The space of unit quaternions can be thought of as the sphere $\mathbb{S}^3 \subseteq \mathbb{R}^4$ so the linear properties can be exploited to define the derivative of a quaternion curve as follows:

$$\dot{q}(t) = \frac{d}{dt}q(t) = \lim_{h \to 0} \frac{q(t+h)-q(t)}{h}$$

As $\mathbb{S}^3$ is a Lie group, the angular velocities of $q(t)$ can be represented as a vector $\Omega(t) \subseteq \mathbb{R}^3$ such that:

$$\dot{q}(t) = \frac{1}{2} q(t) * \begin{bmatrix} 0 \\ \Omega(t) \end{bmatrix}$$

Inverting this equation we obtain that

$$\Omega(t) = \lim_{h \to 0} 2Im(\frac{\bar{q}(t) * q(t+h)}{h})$$

where the imaginary part excludes only the first component, which is always zero in this expression.
As a consequence, $\Omega(t) = 2Im(\frac{\bar{q}(t)*q(t+\delta)}{\delta})$ is a good approximation of the angular velocity for small values of δ.
In order to go back from angular velocity to the unit quaternion time series we can exploit a forward Lie-group Euler method to calculate the next orientation $q(t+\delta)$ from $q(t)$ and $\Omega(t)$, defined as follows:

$$q(t+\delta) = q(t) * e\tilde{x}p(\delta\Omega(t))$$

where $e\tilde{x}p: \mathbb{R}^3 \to \mathbb{S}^3$ is the exponential function in Lie groups, defined as power series. For unit quaternions it has the closed form $e\tilde{x}p(v) = \cos(\frac{1}{2}||v||) + \frac{v}{||v||}\sin(\frac{1}{2}||v||)$ where $v \in \mathbb{R}^3$ and $||\bullet||$ is the standard Euclidean norm (see[5] and[6] for further details).

3 Graphical representation of unit quaternion time series

The component-wise representation is not the only way to visualize unit quaternion time series. In this section, we depict data at hands in two alternative way: axis-angle representation and sterographical projection.
As a representative example, in Figure S2 and Figure S3 the first time series of both the datasets used in the text was represented on the sphere through the axis-angle parametrization, as described in Equation 1. Clearly this representation is limited by the missing representation of angle θ information.

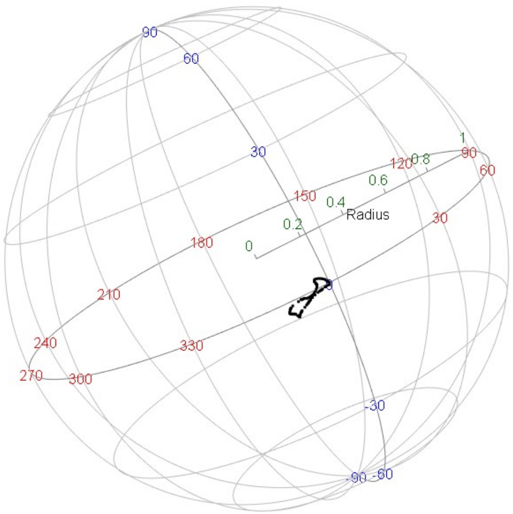

Figure S2. Representation of the first time series in individual gait pattern data.

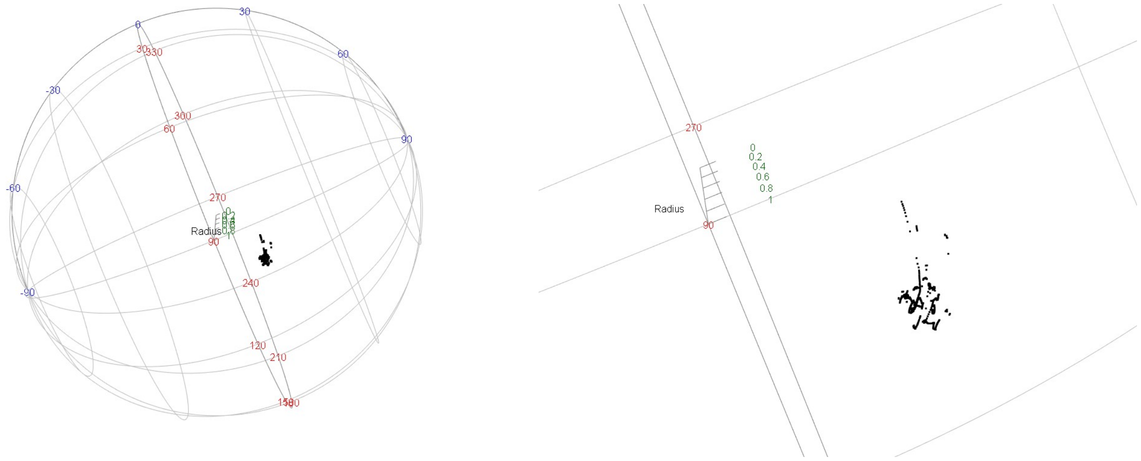

(a) First time series.

(b) Zoom ofthe first time serie

Figure S3. Representation of the first time series in Abdominal Sensor data represented as vector-axis.

In Figure S4 and Figure S5, stereographical projection is adopted to project unit quaternions in the $\mathbb{R}^3$ space, as described in[7].

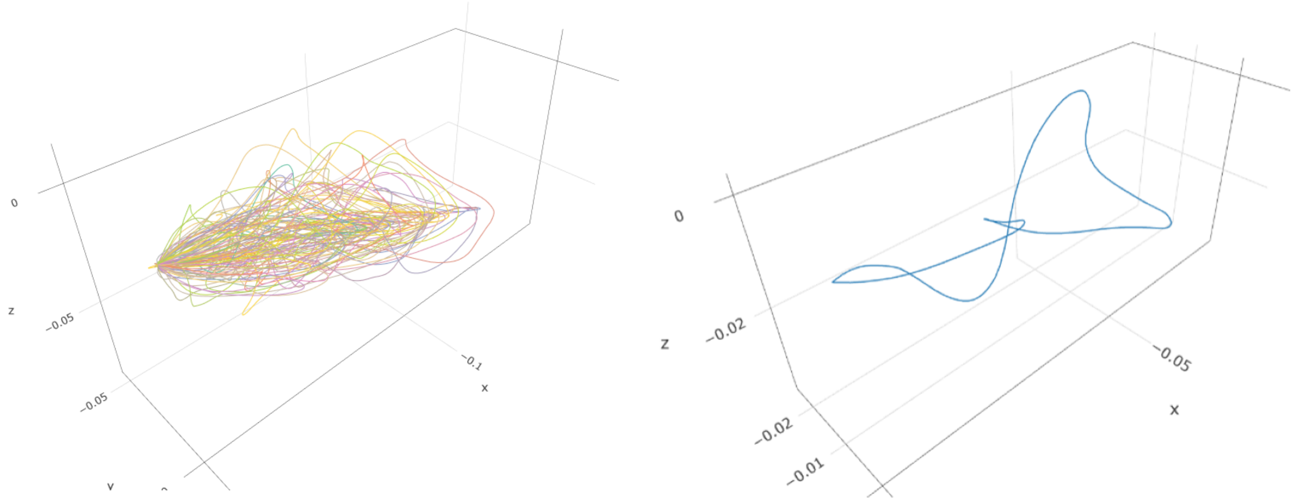

(a) Complete dataset. **(b)** First time serie

Figure S4. Representation of the unit quaternion time series in individual gait pattern dataset as stereographical projection.

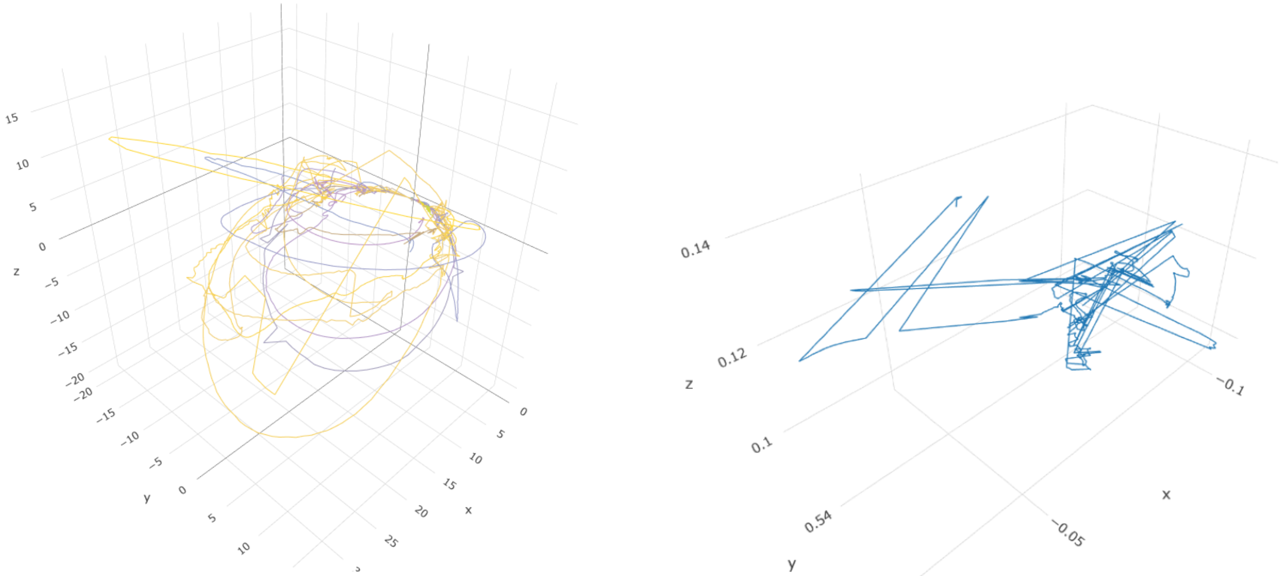

(a) Complete dataset. **(b)** First time serie

Figure S5. Representation of the unit quaternion time series in Abdominal Sensor data. dataset as stereographical projection.

References

1. Dam, E., Koch, M. & Lillholm, M. Quaternions, interpolation and animation (2000).
2. Baker, H. F. Alternants and continuous groups. *Proc. Lond. Math. Soc.* **s2-3**, 24–47, DOI: https://doi.org/10.1112/plms/s2-3.1.24 (1905).
3. Jablonski, B. Quaternion dynamic time warping. *IEEE transactions on signal processing* **60**, 1174–1183 (2011).
4. Shoemake, K. Animating rotation with quaternion curves. In *Proceedings of the 12th Annual Conference on Computer Graphics and Interactive Techniques*, vol. 19, 245—-254, DOI: 10.1145/325165.325242 (1985).

5. Rico-Martinez, J. & Gallardo-Alvarado, J. A simple method for the determination of angular velocity and acceleration of a spherical motion through quaternions. *Meccanica* **35**, 111–118, DOI: https://doi.org/10.1023/A:1004853828657 (2000).

6. Boyle, M. The integration of angular velocity. *Adv. Appl. Clifford Algebr.* **27**, 2345–2374, DOI: https://doi.org/10.1007/s00006-017-0793-z (2017).

7. Terzakis, G., Culverhouse, P., Bugmann, G., Sharma, S. & Sutton, R. On quaternion based parameterization of orientation in computer vision and robotics. *J. Eng. Sci. Technol. Rev. (JESTR)* **7**, 82–93 (2014).
